# The impact of health on economic and social outcomes in the United Kingdom: A scoping literature review

**DOI:** 10.1371/journal.pone.0209659

**Published:** 2018-12-31

**Authors:** Dawid Gondek, Ke Ning, George B. Ploubidis, Bilal Nasim, Alissa Goodman

**Affiliations:** 1 Centre for Longitudinal Studies, UCL Institute of Education, University College London, London, United Kingdom; 2 Department of Quantitative Social Science, UCL Institute of Education, University College London, London, United Kingdom; Cardiff University, UNITED KINGDOM

## Abstract

This is the first review of the evidence, based on longitudinal studies in the United Kingdom, on the association of ill health at any life stage and later social and economic outcomes. The review included a wide range of physical and mental health exposures, both self-reported and objectively measured, as well as social (e.g. life satisfaction) and economic (e.g. employment) outcomes. We searched the Web of Science, key longitudinal datasets based in the UK, major economic journals, Google Scholar and reference lists of relevant publications. The review includes 80 studies. There was strong evidence for the association between early mental health, mainly attention deficit hyperactivity disorder, and lifetime educational, occupational and various social outcomes. Also, both poor physical and mental health in early and middle adulthood, tended to be associated with unemployment and lower socioeconomic status. Among older adults, the evidence quite consistently indicated an association between mental health, chronic conditions, disability/functional limitations, self-rated general health and quality of life, life satisfaction and early retirement. Overall, mental health was consistently found to be associated with a range of social and economic outcomes throughout the lifespan. The evidence for the association between physical health and later outcomes is more inconsistent. A number of methodological challenges need to be addressed, particularly related to causal inference, to produce robust evidence with potential to inform public health policy.

## Introduction

The worldwide economic costs of non-communicable diseases were estimated to be $47 trillion between 2011 and 2030.[1] The burden of non-communicable diseases has been on the rise worldwide, as well as in the United Kingdom.[2] The enormous costs of mental and physical health problems result from increasing demand on health services, as well as from poor health having a deteriorating impact on a range of socioeconomic aspects of life,[1] for instance unemployment and a low educational attainment.[3, 4]

There has been extensive research on the social and economic determinants of people’s health.[5] However, little is understood about the impact of people’s health on the economy and society. There have been few attempts to review comprehensively the evidence of the association between health and socioeconomic outcomes, none of which was conducted specifically in the United Kingdom. Four of those reviews focused exclusively on the link between childhood health and later socioeconomic outcomes (mainly educational attainment and employment-related outcomes), all finding strong evidence for the association.[3, 4, 6, 7] Another review found supportive evidence for the association between poor self-rated health, mental health as well as chronic diseases and exit from paid employment through disability pension, unemployment and early retirement.[8] Due to a rapidly growing body of evidence, an updated comprehensive review is needed, which would investigate the association between poor health occurring throughout the lifespan and a wider range of economic and social outcomes.

In order to inform policy and practice, the evidence of the highest quality needs to be considered. Arguably, the most suitable type of data resources are longitudinal studies as they allow for taking into consideration the temporal relationship between the variables and ensuring that the relationship between health and socioeconomic outcomes is not reversed. Another unique contribution of longitudinal studies, is the availability of time-varying exposures that may confound these associations. This means that they can take into account feedback between the exposure and outcome over time. Hence, longitudinal studies allow for a robust inference about causal relationships.

The UK is particularly privileged in having a range of large, population-representative longitudinal studies. Hence, we aimed to review how longitudinal data from the UK have been used up to date in examining relationships between the health of individuals, and their economic and social outcomes, specifically focussing on three key areas:

(i) the association between ill health during childhood or teenage years (age up to 18-years-old) and outcomes across the life course;
(ii) the association between ill health in early adulthood and middle age (ages 19–49) and later outcomes;
(iii) the association between ill health at older ages (50-years-old or older) on later outcomes.

The main objectives of this scoping review was to assess the amount of evidence (i.e. number of studies), consistency (i.e. if most studied associations between a predictor and outcome were in the same direction) and quality of the evidence, and to understand the further potential for longitudinal datasets in studying the association between the health of individuals and economic and social outcomes, focusing mainly on causal inference methods.

## Methods

We conducted a scoping review, according to the guidelines outlined by the Knowledge to Action research program.[9] The protocol for the study was not published (S1 Text). We considered a wide range of physical health indicators, both self-reported and assessed by health professionals. This included self-rated general health status, disability and functional limitations, chronic conditions or conditions affecting body systems (e.g. cardiovascular, respiratory), sensory limitations, proxies for early development and foetal growth conditions (e.g. height, birthweight), and objective measures of health such as peak flow rate or blood pressure. Likewise, the mental health indicators were broad, including any disorder from DSM-V/ICD-10[10, 11], or earlier versions, and common measures of self-reported psychological distress (e.g. General Health Questionnaire).[12]

The social outcomes of interest were partnership status, family characteristics (including parenting), social networks, social capital, life satisfaction/quality of life and educational attainment. We included a variety of economic outcomes related to employment, socioeconomic status, income and wealth. We did not use any specific definitions of these concepts in order to include a range of methodological and theoretical approaches to studying these outcomes.

### Search strategy

Three concurrent search strategies were used to secure as complete a sample as possible of studies based on UK longitudinal datasets. First, relevant studies were identified through a wide-ranging multidisciplinary search engine, the Web of Science (search took place on 13/02/2017; no publication years limits imposed) using a comprehensive set of search terms for health indicators and social/economic outcomes (S1 Table). Second, manual searches were conducted on key longitudinal data sets based in the UK (13-18/02/2017). In addition, we manually searched key economic journals suggested by experts in the field, the Handbook of Health Economics and the Encyclopaedia of Health Economics. We also searched the OpenGrey Repository for any unpublished studies, for example dissertation theses. Thirdly, we found studies by examining the references and citations made within (and of) other studies included in the review and we searched Google Scholar using the key words for each outcome included in the main search (S1 Table) in combination with broad terms related to health (e.g. health, morbidity). Finally, we consulted experts to identify any other key relevant publications.

### Eligibility criteria and selection process

The first author (DG) initially screened titles and abstracts of the identified citations and subsequently full texts (of the remaining citations) according to the inclusion/exclusion criteria (see S2 Table for reasons for exclusion) ((i) study examined association between any of the health indicators of interest, as an exposure variable, and a social/economic outcome, as an outcome variable; (ii) study was based on the UK-based datasets of interest; (iii) study was published in English)).

### Data extraction and quality assessment

The first author conducted data extraction using a pre-designed form that included information on study sample, health exposure and age at which it was measured, social/economic outcome and age at which it was measured, type of measure of the association (e.g. odds ratio) and its strength from the most adjusted model and the list of covariates adjusted in that model (see Tables A-I in S1 File). Due to heterogeneity in the studies, where the exposure and outcomes were reported at different ages and providing different measures of association (e.g. odds ratio, risk ratio, beta coefficient), the evidence is summarised narratively, focusing on the quality, amount of evidence (i.e. number of studies), consistency (i.e. if most studied associations between a predictor and outcome were in the same direction). The quality of studies was assessed by a co-author (KN) using an adapted version of the Newcastle-Ottawa Quality Assessment Form for Cohort Studies (see Table A in S2 File for the criteria and Table B in S2 File quality appraisal of individual studies).[13] The ‘selection’ (with maximum of three stars) domain included representativeness of the sample, ascertainment of exposure, demonstration that outcome of interest was controlled for or not present at the baseline of a study. Selection of the non-exposed cohort and `comparability`domain were not included as all studies drew the exposed and unexposed samples from the same cohorts. Instead, ‘adjustment’ domain was added: one star was given if a study controlled for relevant confounders (beyond sex, marital and socioeconomic status); two stars were given if a study used causal inference methods (e.g. instrumental variables approach). Within the ‘outcome’ domain (maximum two stars), two categories were included: assessment of outcome and adequacy of follow-up of cohorts. Length of follow-up was replaced by whether studies used longitudinal design, in which exposure preceded outcome (granted with one star). Studies are considered of high quality on following conditions: two or three stars in ‘selection’ domain as well as they must have been somewhat or truly representative for the general population (as we were interested in generalisable findings); one or two stars in ‘adjustment’ domain; at least one star for lack of bias due to loss to follow-up. Studies that were not adequately adjusted or had no points in ‘selection’ domain were considered of poor quality. To sum up, all studies of good quality were longitudinal, somewhat or truly representative for the general population, controlled for a rich set of confounders, and were unlikely to suffer from a bias due to attrition (Table A in S2 File). Whereas, all those of fair quality were somewhat or truly representative for the general population and controlled for a rich set of confounders. Those that did not meet the above criteria were considered of poor quality.

## Results

**Fig 1:**
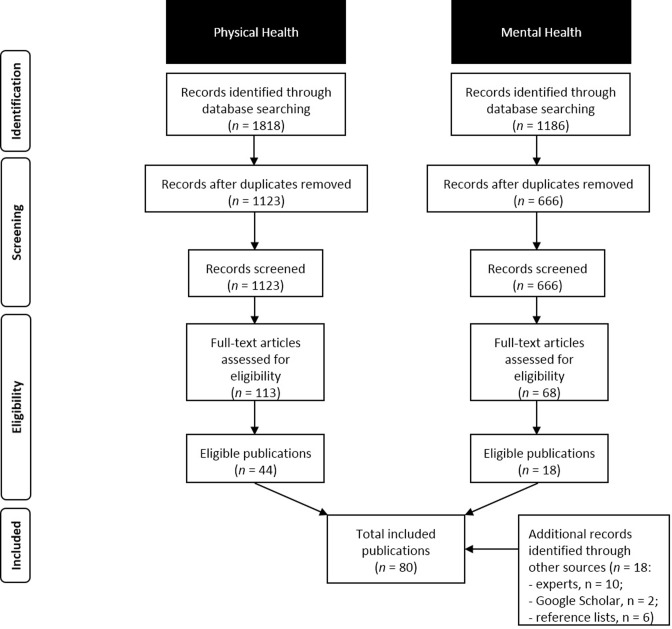
Flow diagram of study selection.

As presented in Fig 1, we identified 1818 citations for physical health exposure and 1186 for mental health exposure. After screening of the titles, abstracts, and subsequently assessing eligibility of full texts, 62 studies were included (see S2 Table for reasons for exclusion). After identifying additional 18 studies, the final sample constituted 80 studies. We present the main findings for three life stages at which ill health occurred (younger age, middle age, older age). For key information from individual studies see Tables A-I in S1 File.

### Life stage 1

This stage included 49 studies, 25 (51%) studies focused on physical health, 15 (31%) on mental health, and nine (18%) on both. Studies were mostly based on the National Child Development Study (NCDS) (n = 24; 49%), the Avon Longitudinal Study of Parents and Children (ALSPAC) (n = 12; 25%), and the 1970 British Cohort Study (BCS70) (n = 9; 18%). Around half of the studies were of good quality (n = 26; 53%; Table B in S2 File), as they used secure records or structured interviews to measure exposure (n = 12; 46%), demonstrated that outcome was not present at baseline or was controlled for (n = 21; 81%) and used record linkage/independent blind assessment to measure the outcome (n = 7; 27%). Half of studies controlled for a rich set of observable confounders, some of which preceded the measurement of health, for instance cognitive ability, parental (e.g. parental education), family (e.g. family size) or household (e.g. household income) characteristics (49%; n = 24). Studies also commonly (n = 12; 25%) took into consideration factors occurring during pregnancy and infancy that may be associated with later health, for instance mother`s smoking, birth complications, birthweight or breastfeeding. Five studies (10%) used methods aiming to account for unobservable confounding variables (e.g. fixed effects models, instrumental variables).

A large proportion of studies (n = 21; 43%; Table B in S2 File) were of fair quality: they tended to measure exposure using secure records or structured interviews (n = 10; 48%), demonstrated that outcome was not present at baseline or was controlled for (n = 2; 10%), were longitudinal (n = 19; 91%), used record linkage/independent blind assessment for ascertainment of outcome (n = 3;14%), and were unlikely to suffer from a bias resulting from a loss of sample due to attrition (n = 13; 14%). Two (4%) studies were of poor quality.[14, 15]

#### Physical health

Within physical health, obesity was the most widely studied condition, and the focus of a considerable volume of good quality evidence (7 out of 25 studies; 28%). The association between obesity or overweight and a number of social outcomes in childhood/adolescence, such as academic performance and attainment,[16–19] and peer problems,[20, 21] has been investigated showing mainly inconsistent findings, depending on the age at which the measurement was taken or methods used. Studies tended to take advantage of the longitudinal nature of the data by considering BMI trajectories throughout childhood,[22] and persistence of obesity into adulthood.[18, 23, 24] In addition, quasi-experimental methods, involving instrumental variables and the constant nature of certain characteristics over time (fixed effects models), were used as a means of inferring causality.[17] Scholder and colleagues attempted to address the inconsistency in findings on the relationship between child adiposity (or BMI) and educational outcomes by comparing results from the most commonly used methodological approaches, such as ordinary least squares (OLS), fixed effects and a set of instrumental variables, and concluded that fat mass is unlikely to be causally related to academic achievement in adolescence.[17] Three studies produced mixed findings on the association between obesity in childhood/adolescence and wages as well as employability prospects in the early and mid-adulthood. One study did not find evidence for the association of obesity at age 10 and unemployment at age 30 after adjusting for a range of confounders, such as maternal education, social class in childhood and adulthood,[18] one–equally well-adjusted–study supported the association between obesity at age 16 and lower hourly salary at age 23 for females only (on average by £5.3 CI 95% 8.3 to 2.3 compared to non-obese females)[23] and one provided supportive evidence for the association between obesity and salary at age 30 for both females and males if the obesity was persistent throughout childhood and adolescence.[24]

The association between a number of chronic or body systems conditions, for instance related to cardiovascular and respiratory functioning, and later educational and occupational attainment was also investigated. The evidence, of good or fair quality, was mixed when the association of individual conditions, such as eczema, asthma/wheezy bronchitis, epilepsy, meningitis, or amblyopia and later outcomes was considered (see Tables A-I in S1 File).[25–30] Studies of good quality demonstrated that persistently poor health, measured as long-standing limiting illness, chronic or systems conditions was rather consistently associated with worse educational and occupational outcomes.[19, 31–35] In terms of other social outcomes, one study, of good quality, found the association between the number of chronic health conditions at age seven as well as having a physical handicap or disabling condition between age seven and eleven and lower adult life satisfaction between age 33–50.[36]

There was also fair quality evidence for the association of higher height and more positive later educational and occupational outcomes.[37–39] However, these associations tended to be explained by cognitive ability,[40] a combination of cognitive and non-cognitive abilities (e.g. locus of control, management skill)[41, 42] or social capital.[43]

There was also some evidence driven by foetal origins hypothesis stating that poor foetal conditions, for instance related to nutrition–reflected by low birthweight and height–may predispose an individual to poor health in the adulthood, which in turn can lead to worse social or economic outcomes.[44] Several studies, of good quality, found a relationship between low birthweight and later outcomes, particularly with lower educational attainment,[19, 31, 45], unemployment,[45] and lower life satisfaction,[36]. Whereas, other studies–also of good quality–failed to support the hypothesis on the association between low birthweight and employment stability,[35] intergenerational social mobility,[35] attained social class,[46] wages[45] or produced mixed findings on educational performance (i.e. the association was found with math but not reading skills).[32] One study–of good quality–which focused on weight faltering, rather than birthweight, found an association with greater odds of developing peer relationships at age eight (OR = 1.26, Cl95% 1.01–1.56), but not with self-esteem (OR = 0.96, 95% 0.77–1.20) or being bullied (OR = 1.17, Cl95% 0.89–1.53).[47]

#### Mental health

As shown by evidence of mainly good quality (6 out of 8 studies, 75%), a range of economic outcomes appear to be associated with prior poor mental health, for instance, those who experienced psychological distress, attention deficits or externalising behaviour problems in their childhood or adolescence may be more susceptible to unemployment, lower earnings or lower occupational class.[19, 35, 48–53] In addition, the evidence–equally split across fair and good quality studies–suggests a fairly consistent association between poor early mental health and lower educational attainment (e.g. attainment at GCSE), such association was found for externalising behaviour problems,[50, 51, 54–56] attention-deficit/hyperactivity,[54, 55, 57] psychological distress[19, 58] and self-harm with suicidal intent.[59] In terms of other social outcomes, externalising behaviour problems were found to be associated with poorer relationships and family problems.[51] However, as shown by studies good and fair quality, there was no evidence for relationship between poor mental health in adolescence and later parental practices, such as parental interest or stimulation.[35, 60, 61] In a similar vein, psychological distress was found, in studies of good quality, to be associated with lower likelihood of being married or in a relationship by age 50, [35] but psychological distress was not associated with breakdown of first partnership.[61] Finally, one fair quality study found evidence for an association of early emotional problems (age 16) on lower life satisfaction in older age (age 60–64).[62]

The evidence–of fair quality–on the association between poor mental health indicators, such as psychotic symptoms (OR = 1.12, Cl95% 0.43–2.91) [63], pain-related anxiety (females: B = 0.228, p<0.001; males: B = 0.047, p>0.05) [14] and peer problems, appeared to be inconclusive.

### Life stage 2

We identified 18 studies for life stage 2, most of which came from NCDS (n = 8; 44%). Eleven (61%) of the included studies focused exclusively on physical health, while four (22%) included mental health predictors and three (17%) included a combination of both physical and mental health predictors. Most studies were of good quality (n = 13; 72%; Table B in S2 File), which used secure records or structured interviews to measure exposure (n = 5; 39%), demonstrated that outcome was not present at baseline or controlled for it (n = 3; 23%) and used record linkage to measure the outcome (n = 1; 8%). Most of the studies (n = 14; 78%) took advantage of the longitudinal nature of the data, such as an ability to control for a wide range of confounding factors occurring across the lifespan (e.g. birth weight, cognitive ability in addition to education, parental variables) (see Tables A-I in S1 File). In addition, three (17%) of the included studies used instrumental variables to account for unobserved confounding.[64–66]

Five (28%; Table B in S2 File) studies were of fair quality: they measured exposure using secure records or structured interviews (n = 3; 60%), demonstrated that outcome was not present at baseline or controlled for it (n = 3; 60%), were longitudinal (n = 3; 60%), used record linkage/independent blind assessment for ascertainment of outcome (n = 1; 20%), and were unlikely to suffer from a bias resulting from a loss of sample due to attrition (n = 4; 80%). One (6%) study was of poor quality.[15]

#### Physical health

Economic outcomes, such as unemployment or lower socioeconomic status were found–in studies of good and fair quality–to be associated with prior poor health, measured as disability due to an accident,[66] musculoskeletal symptoms,[67] poor vision[68, 69] and a combination of self-reported general health and health limitations.[70] Furthermore, those in poor self-rated health had also over fivefold higher odds of receiving incapacity benefits (OR = 5.5, Cl95% 2.3–12.9), [71] and had higher odds of downwards intergenerational (males: OR = 1.40, Cl95% 1.03–1.91; females: OR = 1.63, 95% 1.18–2.21) and intragenerational (males: OR = 1.94 Cl95% 1.43–2.64; females: OR = 1.65, Cl95% 1.25–2.16) mobility; [72] and poor visual functioning appeared to be associated with lower socioeconomic status (OR = 1.23, 95% 1.08–1.41), lower quality of life (OR = 2.39, 95% 1.17–4.86), higher odds of not being married (OR = 1.67, 95% 1.31–2.12).[68] There was also good quality evidence on the association between obesity and greater unemployment and lower earnings among women.[64, 65] However, the results were inconsistent across different methodological approaches, with studies employing instrumental variables providing no evidence for a causal relationship.[69, 73]

In terms of social outcomes, those with a disability were found to be less likely to be in a relationship or married and have children, as well as more likely to divorce, however the findings came from a poor quality study.[15] Also, lower social participation, measured as taking part in general elections, appeared to be associated with self-rated poor health.[74] On the other hand, self-rated general health was not found–in a good quality study–to be associated with quality of various aspects of parenting (e.g. warmth, support, rejection and control).[75]

#### Mental health

Psychological distress was found in studies of good quality to be associated with a range of economic outcomes, including long term sickness absence,[67] lower job security and more negative job characteristics [52] as well as lower household income.[58] As far as social outcomes were concerned, psychological distress was associated with higher risk of unmet residential mobility preferences in a good quality study,[76] however there was mixed fair quality evidence of its association with social participation, measured as taking part in general elections.[74] Good quality evidence was also produced on the association between maternal depression and later outcomes, showing evidence on the link between maternal depression with low quality of parenting but not on risk of not being in education, training or employment.[75, 77]

### Life stage 3

In this stage 20 studies were included, out of which 15 (75%) were based on the English Longitudinal Study of Ageing (ELSA). A majority of the studies focused on physical health (n = 16; 80%), one (5%) on mental health and three (15%) both on mental and physical health. Nearly half of the studies were of good quality (n = 9; 45%; Table B in S2 File): three (33%) used secure records or structured interviews to measure exposure, all of them demonstrated that outcome was not present at baseline or controlled for it, and all relied on self-reported measures of outcome. Five (25%) studies were of fair quality: all of them measured exposure using self-reports, four (80%) demonstrated that outcome was not present at baseline or controlled for it, three (60%) were longitudinal, all used self-reports to measure outcome, and two (40%) were unlikely to suffer from a bias resulting from a loss of sample due to attrition. Four (20%; Table B in S2 File) studies were of poor quality.

The included studies tended to be well-adjusted for baseline confounders (n = 13; 65%). However, as most studies used the ELSA, which started following individuals aged 50 or older, information on confounders occurring throughout the lifespan (e.g. early health or cognitive ability) was not available. Thus, any attempts to control for factors occurring prior to health measurement would focus mainly on maximum educational attainment. Also, one (5%) study attempted to control for unobservable confounders in order to test a causal relationship between acute health shock (i.e. the first onset of myocardial infarction, stroke or cancer) and later lower labour market participation.[78]

#### Physical health

The evidence, of mixed quality, fairly consistently indicated that participants tended to report worse quality of life and life satisfaction if they had suffered from disability,[79, 80] chronic conditions,[73, 79, 81, 82] cancer,[83] coronary heart disease[84], lung problems,[85] higher BMI among females,[85] having a limited long-standing illness, [80, 82] having a difficulty with a daily activity of living,[73, 82], poor self-reported general health,[36] and poor vision.[41, 68, 69] Poor self-reported general health and vision were also found–in studies of good quality–to be associated with lower social participation (e.g. being a member of a club/society),[69, 86] and poor self-rated oral health was associated with having less close social ties.[87] However, there were also two studies of good quality that found no association between objectively measured health, such as blood pressure and BMI[85] or BMI and waist circumferences[82] and quality of life.

Among economic outcomes, early retirement was rather consistently associated–in mixed quality studies (with 4 out 7; 57% of good quality)–with having experienced poor health, operationalised as a variable combining multiple indicators (e.g. disability, chronic conditions),[88, 89] disability or functional limitations,[90, 91] self-reported poor health,[89, 91] acute health shock (the first onset of myocardial infarction, stroke or cancer)[78, 88, 89, 92] and diabetes.[93] Nonetheless, Rice and colleagues, in a good quality study, did not find an association between obesity, high blood pressure, having a heart problem and early retirement.[91] Among other oucomes, there was no association found between deterioration in self-reported vision and earnings.[69]

#### Mental health

Only four studies, two of good and two of fair quality, investigated the association between poor mental health, operationalised as psychological distress or depression, and later outcomes. The evidence consistently indicated that poor mental health was associated with lower quality of life,[73, 80] life satisfaction, [62] and early retirement.[91]

## Discussion

### Life stage 1

Studies investigating the association between specific physical health conditions and later outcomes produced mixed results. For instance, obesity or overweight, were the most commonly studied health exposures, resulting in a considerable volume of high quality evidence, which was highly inconsistent due to methodology used or age at which measurement was taken. These inconsistencies were also seen in non-UK based evidence. For instance, there were no associations found between childhood health conditions, such as middle-ear disease[94] or amblyopia,[95] and socioeconomic status, employment status or educational outcomes in adulthood. However, low birthweight and height as markers of childhood health were found to be associated with poorer educational outcomes[37, 96, 97] and lower socioeconomic status even when compared to siblings of the participants.[96, 98]

There was strong evidence for an association between early poor mental health and education, this was also emphasised in a previous, worldwide overview of the literature.[3] Specifically, ADHD or inattention were consistently shown as being associated with lower academic attainment, as indicated by a systematic review of population-based prospective studies[7] and other individual studies conducted in New Zealand,[99–101] USA[102] and Canada[102, 103]. As shown by studies using siblings comparisons, the negative association between ADHD and education is likely to be causal in nature and appears to have larger effects than depression or conduct disorders.[102, 104] Other mental health conditions, such as depression, conduct disorders, were also revealed to be associated with worse educational outcomes, as well as social outcomes, such as relationship problems.[105, 106] In addition, poor early mental health was found to be associated with worse employment prospects and earnings, also when compared to siblings and when physical health conditions or self-rated health were accounted for.[107, 108]

### Life stage 2

Overall, the relationship between physical health and economic outcomes was more consistent compared to the association with social outcomes. These findings were consistent with the evidence from outside of the UK. For instance, Van Rijn and colleagues found that self-perceived poor health, chronic conditions and poor mental health were risk factors for transition into disability pension and unemployment.[8] Having permanent health conditions was also found to be associated with lower wages, particularly among women.[109] Consistently with the evidence outside of the UK, obese women but not men tended to have lower earnings [110] and reduced chances of career progression.[111] However, as it was the case in the UK studies, when instrumental variables were used there was no evidence for a causal relationship.[110]

There was strong and consistent evidence showing negative association between mental health and social/economic outcomes. Similar findings were also obtained by the World Health Organization (WHO) World Mental Health Survey, which was a cross-sectional population survey conducted in 27 countries, early-onset mental disorders were associated with significantly reduced household income due to low personal earnings (increased unemployment, decreased earnings among the employed) and spouse earnings (decreased probabilities of marriage and, if married, spouse employment and low earnings of employed spouses).[112]

### Life stage 3

The evidence on the association between poor health and social outcomes was somewhat more consistent compared to that of economic outcomes. The evidence consistently indicated a negative association between poor mental health and quality of life,[73, 80] life satisfaction, [62] and early retirement. [91] Consistent with the evidence from the UK, the systematic review conducted by van Rijn and colleagues [8] showed that self-perceived poor health and mental health problems, but not chronic diseases, were risk factors for early retirement. Poor health due to a number of chronic conditions, such as diabetes, asthma, cancer, heart disease, stroke was also associated with lower economic productivity and wealth.[113–115]

### Establishing causality

In this section, we discuss the limitations of the current evidence in establishing causality. A vast majority of the studies have relied on multivariable adjustment with regression-based methods, which identify unbiased estimates of the causal relationships between health and later outcomes only on the assumption that there is no selection on unobserved characteristics which are correlated with both the health exposure and the later outcomes in question. Many studies, particularly within stages 1 and 2, tended to account for a wide range of relevant confounding factors due to availability of rich data on the entire lifespan within birth cohort studies. It was a common practice to adjust for factors that preceded both health measurement and later outcomes, for instance parental characteristics (education, health behaviours), birthweight, birthweight complications or cognitive ability. Such variables are particularly likely to be associated with both the quality of health (the predictor) and later social and economic outcomes, thus accounting for them potentially provides an estimate of the causal association between health and later outcomes. Nonetheless, multivariable adjustment with regression-based methods does not account for unobserved sources of confounding. Thus, depending on the application in question, regression-based methods may mask a true association due to unobserved factors.

There have been a few attempts uncovered by our review to account for unobserved factors that may have a confounding effect, thus directly testing causal relationships under less stringent assumptions. These included fixed effects models,[17] and instrumental variables models.[17, 24, 64, 65] These methods were mainly limited to investigating the impact of obesity (or BMI) on later outcomes. Thus, further research in the field using these approaches is needed, however the limitations of these methods need to be considered.

As emphasised by Scholder and colleagues,[17] each of these approaches is based on different assumptions, which may not be necessarily valid in this context. Fixed effects models account for confounding effect of any unobserved factors, which do not vary over time (time-invariant) reducing the amount of potential bias. Nonetheless, this comes at a price of not being able to include time-invariant observed factors, which for instance, may help to understand the mechanisms explaining the association between health and later outcomes. Fixed effects models operate under the assumption of strict exogeneity, which prohibits feedback from past outcomes to current exposures and current outcome to future exposures, which may be violated in a longitudinal study. Furthermore, there may also still be time varying confounding factors (‘non-random shocks’) which lead to biased estimates of causal relationships, even when fixed effects are used.[116]

Siblings comparisons is another method that has been used, in which an individual with a given exposure, is compared to a sibling without such an exposure.[117] The strength of this approach is that it allows for controlling for a range of genetic and environmental factors that may be unobserved and which are shared within the same families (e.g. socioeconomic characteristics, attitudes and values). The downside of this method is reduced generalisability of findings, as siblings who are differentially exposed to critical risk factors may be not representative of all siblings in the population, as well as siblings may differ in an important way from singletons.[118] In addition, information on siblings is not as widely available as for the main participants.

One of the approaches that can remove the problem of unobserved confounding involvs using instrumental variables. Nonetheless, identifying a valid instrument that meets all the relevant assumptions is problematic. For instance, Scholder and colleagues used three sets of instruments for child adiposity, all of which have been applied in the literature: mother’s pre-pregnancy BMI, the child’s lagged adiposity categories, and the child’s genetic markers. As shown, the non-genetic instruments were likely to be associated with several child and family background characteristics that were also associated with children’s educational outcomes, thus they did not meet the exclusion restriction criteria (i.e. the instrumental variable is only related to the outcome via its effect on the predictor).[17] There are also other methods testing causal relationships, which have recently been gaining in prominence and not been used in studying health–socioeconomic outcomes association, for instance the negative controls approach or ‘Mendelian randomisation’.

In the negative controls approach the association of interest is compared with another similar association, which has a comparable confounding structure, but includes a predictor or outcome for which there is no biologically plausible mechanism for causation.[119] As an example, factors associated with infant development in utero, such as mother`s health behaviours, have been compared with the same exposures in fathers, which cannot have a direct intrauterine effect.[120] Another approach that provides a promising avenue for further research is a range of methods using sensitivity analyses, where the effect of hypothetical (unobserved) confounders is simulated and accounted for.[121] This allows for estimating the amount of confounding that would need to be present to alter the obtained association hence helping to judge confidence in the findings.

Another method, which have recently been gaining in prominence and not been used in this context is the ‘Mendelian randomisation’. It utilises individual`s genetic predisposition (a genetic variant) as a proxy for the predictor, which can be used as an instrumental variable. Genetic variants are considered good candidate instrumental variables as the genotype is assigned randomly (given the parents’ genes) at meiosis and is not mutable by environmental factors.[122] The UK-based longitudinal studies (ALSPAC, ELSA, NCDS, NSHD) benefit from genetic data and the advantage of this approach could be taken in this context. Although application of the approach is limited by the knowledge of genetic variants associated with specific health conditions, large-sample genome-wide studies are increasing continuously generating information on genetic instruments, for instance associated with ADHD.[123] However, it has been shown that even in the case of genetic instruments violations of the exclusion restriction may occur in the form of pleiotropy and linkage disequilibrium.[124] *Pleiotropy* is the phenomenon whereby a single gene may influence several traits, or when a genetic polymorphism under study might have pleiotropic effects that influence confounding factors like smoking or alcohol. *Linkage disequilibrium* refers to the association between alleles at different loci across the population, as is the case when loci are physically close on the chromosome and thus tend to be inherited together, or may be due to other reasons such as natural selection, assortative mating and migration.

### Triangulation of evidence from different contexts

Furthermore, comparison of studies conducted in various contexts, potentially having different confounding structures (e.g. studies in different time periods, countries, populations), also contributes to establishing if there is a causal relationship between predictor (health) and outcome (social/economic).[125] For instance, Jivraj and Nazroo investigated if having poor health was associated with low life satisfaction and quality of life both in England and the USA, and they found that there was a strong association in both countries, despite the differential effect of confounding factors, such as education or marital status.[79]

### Challenge of measuring health

Within stage 2 and stage 3, researchers have relied heavily on self-reported or self-rated measures of health, mainly general health status, disability or chronic conditions (in contrast, health measures in stage 1 mainly came from medical examinations). This can result in a bias as survey respondents may report their health differently, for instance, depending on their understanding of the questions, expectation of their health or due to health incentives related to receiving welfare benefits.[126] Often these discrepancies in reporting are systematic, differing between genders or across the spectrum of socio-economic status,[127] which may affect the measurement of the association between health and later outcomes. Main approaches to mitigate the risk of bias due to those challenges were creating a cumulative health variable including multiple health indicators or using objective measures of health (e.g. biomarkers). These methods, however, have been rarely implemented in studying the link between poor health and later social/economic outcomes with the UK-based data.

### Cross-cohort comparisons

Comparisons across cohorts were relatively rare, mostly occurring within stage 1, hence there is a need for more cross-cohorts studies testing whether associations are changing across generations (given major generational shifts in health and socio-economic status now occurring).[34, 36, 40, 48] Also, multiple birth cohorts (e.g. British birth cohorts) could be combined in single study to investigate the association between health and later outcomes in early/middle adulthood, as it has been already done for cognitive ability and later outcomes[128] and socioeconomic status and subsequent health.[129]

### Mechanisms explaining associations

There is a need for a greater number of studies aiming at explaining mechanisms translating poor health into later outcomes, which would be crucial for policy implications. Unfortunately, such attempts have been very rare. For instance, it has been shown that the association between height and later occupational outcomes tended to be explained by cognitive ability,[40] a combination of cognitive and non-cognitive abilities (e.g. locus of control, management skill)[41, 42] or social capital.[43] Also, health and education outcomes in adolescence were studied as potential pathways between childhood psychosocial development and occupational outcomes.[42]

### Trajectories across the lifespan

There is a lack of evidence on the association between health trajectories across the life course and subsequent outcomes. There are some exceptions, such as work focusing on trajectories of obesity from childhood to adolescence or middle adulthood and later outcomes.[18, 22–24] While Lee and Jackson captured how qualitatively different health trajectories, for instance health deteriorating in childhood/adolescence and improving during adulthood, are associated with lower occupational attainment.[33] Overall, however, there is significant space for more research of this kind, as also noticed by other researchers (e.g. Power and colleagues[130]). Studying trajectories would also contribute to uncovering mechanisms through which health is associated with later outcomes. For instance, it would help to understand if the association between a given health problem and a later outcome differs according to the timing of its occurrence, providing strong implications for health interventions.[131]

### Limitations of this review

The main limitation of this study is that only one database was searched (the Web of Science), however this was compensated by thorough screening of reference lists, study specific search engines and consultations with experts. This process was cyclical until new studies no longer were identified, thus we believe that there is a low risk of studies being missed. Another limitation is that only one reviewer conducted the screening, eligibility assessment and quality appraisal. Nonetheless, the eligibility and quality appraisal criteria were clearly defined, and numerous research group meetings took place during which any uncertainties were discussed.

## Conclusion

The evidence suggests there are likely to be substantial social and economic benefits to be gained across people’s lives if effective interventions can be found to maintain and enhance people`s health. Such evidence of the social and economic value of health can be expected to strengthen the case for broad investment in the maintenance and improvement of health through action on the wider determinants. Nonetheless, the evidence is still highly inconsistent (particularly for physical health exposures) and a number of methodological challenges need to be addressed before the existence of causal relationships between health exposure measures and social/economic outcomes can be inferred with confidence, providing basis for policy recommendations. While the use of causal methods in examining the role of health in later social and economic outcomes has been rare, we suggest that future research should employ these where possible.

## Supporting information

S1 Table Search strategy. (DOC)

S2 Table Reasons for exclusions of full texts. (XLSX)

S1 File Key information from included studies. (DOC)

S2 File Quality appraisal tool and quality appraisal of individual studies. (XLS)

S1 Text Study protocol. (DOCX)
