# Supplementary material for: The impact of health on economic and social outcomes in the United Kingdom: A scoping literature review
Source: PLoS One. 2018 Dec 31;13(12):e0209659. doi: 10.1371/journal.pone.0209659 (PMC6312330; doi:10.1371/journal.pone.0209659)
Supplement: S1 Table — (DOC) [file pone.0209659.s001.doc]

| S1 Table. | | |
| --- | --- | --- |
| **Mental Health** | (“emotional problem*” OR “mental health” OR “mental disorder*” OR “mental illness” OR “psychiatric disorder*” OR wellbeing OR “mentally ill” OR well-being OR distress OR "neurodevelopmental disorder*" OR autism OR Asperger's OR ADHD OR "Attention Deficit Hyperactivity Disorder" OR ADD OR "attention deficit disorder" OR dyslexia OR "learning disability" OR Tourette`s OR coprolalia OR "Psychotic Disorder*" OR hallucination* OR paranoia OR paranoic OR paranoid OR delusion* OR delusional OR schizophrenia OR schizophrenic OR Schizoaffective OR bipolar OR manic OR mania OR depression OR depressed OR depressive OR "panic disorder" OR "panic disorder" OR agoraphobia OR phobia OR anxiety OR anxious OR worry OR worried OR fidgeting OR "obsessive-compulsive disorder" OR OCD OR helpless OR helplessness OR compulsion* OR "post-traumatic stress" OR PTSD OR "personality disorder*" OR abuse OR trauma OR grief OR bereavement OR "domestic violence" OR "intimate violence" OR "Dissociative Disorder*" OR Depersonalization OR Depersonalisation OR "Dissociative Identity Disorder" OR "Multiple Personality Disorder" OR "Somatic Disorder*" OR hypochondriac OR "Conversion Disorder" OR hysteria OR "Eating Disorder*" OR anorexia OR bulimia OR binging OR purging OR "sleep disorder*" OR narcolepsy OR insomnia OR cataplexy OR "Impulse Control Disorder*" OR "Oppositional Defiant Disorder" OR "conduct disorder" OR kleptomaniacs OR pyromaniacs OR "personality disorder*" OR “Negative Emotionality” OR Detachment OR Psychoticism OR Aggressiveness OR aggressive OR aggression OR Disinhibition OR hostile OR hostility OR impulsivity OR impulsive OR “risk taking” OR risk-taking OR “risk taker” OR “emotional lability” OR self-harm OR “self harm” OR separation OR separated OR borderline OR obsessive-compulsive OR “obsessive compulsive” OR avoidant OR avoidance OR withdrawn OR withdrawal OR anhedonia OR schizotypal OR antisocial OR narcissism OR narcissist OR narcissistic OR grandiose OR grandiosity OR Schizoid OR “not otherwise specified” OR “other specified disorder” OR “unspecified disorder” OR anger OR “emotional adjustment” OR “emotional maladjustment” OR hyperactivity OR “attention deficit disorder” OR “behavioural problem*” OR “behavioural disorder*” OR internali* OR externali* OR suicid* OR neurotic OR “mental retardation” OR “psychological development” OR dementia OR Alzheimer) AND | |
| **Studies** | (“Millennium Cohort” OR “Next Steps study” OR “Next Steps cohort” OR “Longitudinal Study of Young People in England” OR “1970 British Cohort” OR “British birth cohort” OR “National Child Development Study” OR “National Survey of Health” OR “English Longitudinal Study of Ageing” OR “Understanding Society” OR “British Household Panel Study” OR “Avon Longitudinal Study of Parents” OR “Life Opportunities Survey”) AND | |
| **Association** | (Association OR associated OR “associative relationship” OR correlation OR correlated OR relationship OR related OR causation OR causal OR "causal inference" OR predictors OR predicting OR predictive OR effect OR effects OR impact OR exposure OR exposed) AND | |
|  | **Key terms specific for each social and economic factor** | **Hits:** |
| **Partnership status** | (“partnership status” OR married OR marriage OR “marital status” OR partner) | 81 |
| **Family** | (“family” OR “familial life” OR “family life” OR “family conflict” OR “family ties” OR “quality of family life” or “marital conflict” OR “Lone parent” OR “lone parenthood”) | 187 |
| **Social Network** | (“social network” OR friend OR friends OR friendship OR “social contact” OR peer OR peers OR lonely OR loneliness OR “quality of relationship” OR “quality of relationships” OR “network structure”) | 114 |
| **Social capital** | (“social capital” OR “social cohesion” OR “neighbourhood cohesion” OR “neighborhood cohesion” OR “informal social control” OR “collective efficacy” OR “neighbourhood disorder” OR “social disorganization” OR “social disorganisation” OR "social organization" OR "social organisation" OR "community cohesion" OR "community participation" OR "social support" OR "social participation" OR trust OR "emotional support" OR "psychosocial support" OR "community capital" OR "neighbourhood cohesion" OR "neighborhood cohesion" OR "collective efficacy") | 42 |
| **Life Satisfaction** | (“life satisfaction” OR “satisfaction with life” OR “quality of life” OR “personal satisfaction” OR “life dissatisfaction”) | 77 |
| **Education** | (education OR educational OR achievement OR “educational attendance” OR “educational attainment” OR “educational level” OR “educational background” OR “education failure” OR “level of education” OR “years of education” OR “academic attainment” OR “academic achievement” OR “academic background” OR qualification) | 230 |
| **Economic factors (employment, income, wealth)** | (employment OR employed OR unemployed OR unemployment OR career OR “career mobility” OR job OR jobs OR jobless OR joblessness OR work OR income OR wage OR salary OR pay OR earning OR earnings OR productive OR productivity OR “quality of employment” OR wealth OR “asset accumulation” OR savings OR “assets accumulated” OR poor OR poverty OR savings OR “job satisfaction” OR “socioeconomic status” OR “socio-economic status” OR economic OR stature OR absenteeism OR presenteeism OR “work performance” OR “job performance” OR “Labour market” OR “labour market attachment” OR “Worklessness” OR “workless household”) | 455 |
| S1 Table. (cont.) | | |
| **Physical Health** | TS=(“self-rated health” OR SRH OR “health status” OR “general health” OR health OR healthy OR frail OR frailty OR morbid OR morbidity OR “health outcome*” OR disable OR disability OR “functional limitation” OR “functional limitations” OR “functionally limited” OR “functionally impaired” OR “functional impairment” OR “physically impaired” OR “physical impairment” OR “instrumental activities of daily life” OR IADL OR “activities of daily life” OR ADL OR audiometry OR laterality OR co-ordination OR coordination OR chronic OR cardiometabolic OR hypertension OR diabetic OR diabetes OR obese OR obesity OR angina OR vascular OR angina OR “myocardial infarction” OR musculoskeletal OR arthritis OR osteoporosis OR fall OR fracture OR respiratory OR asthma OR bronchitis OR stroke OR cancer OR eczema OR epileptic OR epilepsy OR convulsion* OR seizure* OR “cerebral palsy” OR “ventilatory function” OR “peak flow rate” OR wheez* OR ill OR illness OR disease OR “blood pressure” OR systolic OR diastolic OR pulse OR cholesterol OR height OR *weight OR “heap circumference” OR “head circumference” OR “waist circumference” OR “body mass index” OR BMI OR “infectious disease*” OR “Tuberculosis” OR “bacterial disease*” OR Infection* OR “sexually transmitted” OR spirochaetal OR chlamydia OR chlamydial OR Rickettsiosis OR Rickettsioses OR “viral infection*” OR “viral fever*” OR haemorrhagic OR haemorrhage OR hepatitis OR “Human immunodeficiency virus” OR HIV OR mycosis OR mycoses OR Protozoal OR Helminthiasis OR Helminthiases OR Pediculosis OR acariasis OR infestation* OR “parasitic disease*” OR neoplasm OR neoplasia OR neoplasmic OR anaemia OR anaemic OR “Coagulation defect*” OR purpura OR haemorrhagic OR haemorrhage OR immune OR Inflammatory OR atrophies OR Extrapyramidal OR Demyelinating OR paroxysmal OR “plexus disorder*” OR Polyneuropathy OR Polyneuropathies OR “myoneural junction” OR “neuromuscular junction” OR “Cerebral palsy” OR “paralytic syndrome*” OR “Other disorders of the nervous system” OR Meningitis OR Encephalitis OR myelitis OR encephalomyelitis OR Intracranial OR intraspinal OR atrophy OR atrophies OR “degenerative disorder*” OR neurological OR “thyroid gland” OR “endocrine gland*” OR malnutrition OR "metabolic disorder*" OR parkinson OR parkinsonism OR parkinson`s OR dystonia OR basal ganglia OR Huntington OR “Huntington's disease*” OR "rheumatic fever" OR "hypertensive disease*" OR ischaemic OR pulmonary OR cerebrovascular OR Influenza OR pneumonia OR hernia OR Arthropathy OR Arthropathies OR polyarthropathy OR polyarthropathies OR Arthrosis OR “joint disorder*” OR Dorsopathy OR Dorsopathies OR Spondylopathy OR Spondylopathies OR Chondropathy OR Chondropathies OR “Glomerular disease*” OR “Renal failure” OR “renal disease*” OR Urolithiasis OR “kidney disorder*” OR “kidney disease*” OR "endocrine disease*" OR "nervous system disease*" OR "circulatory system" OR "respiratory system" OR "digestive system" OR "musculoskeletal system" OR "genitourinary system") | |
| **Studies** | (“Millennium Cohort” OR “Next Steps study” OR “Next Steps cohort” OR “Longitudinal Study of Young People in England” OR “1970 British Cohort” OR “British birth cohort” OR “National Child Development Study” OR “National Survey of Health” OR “English Longitudinal Study of Ageing” OR “Understanding Society” OR “British Household Panel Study” OR “Avon Longitudinal Study of Parents” OR “Life Opportunities Survey”) | |
| **Association** | (Association OR associated OR “associative relationship” OR correlation OR correlated OR relationship OR related OR causation OR causal OR "causal inference" OR predictors OR predicting OR predictive OR effect OR effects OR impact OR exposure OR exposed) | |
|  | **Key terms specific for each social and economic factor** | **Hits:** |
| **Partnership status** | (“partnership status” OR married OR marriage OR “marital status” OR partner) | 115 |
| **Family** | (“family” OR “familial life” OR “family life” OR “family conflict” OR “family ties” OR “quality of family life” or “marital conflict”) | 242 |
| **Social Network** | (“social network” OR friend OR friends OR friendship OR “social contact” OR peer OR peers OR lonely OR loneliness OR “quality of relationship” OR “quality of relationships” OR “network structure”) | 114 |
| **Social capital** | (“social capital” OR “social cohesion” OR “neighbourhood cohesion” OR “neighborhood cohesion” OR “informal social control” OR “collective efficacy” OR “neighbourhood disorder” OR “social disorganization” OR “social disorganisation” OR "social organization" OR "social organisation" OR "community cohesion" OR "community participation" OR "social support" OR "social participation" OR trust OR "emotional support" OR "psychosocial support" OR "community capital" OR "neighbourhood cohesion" OR "neighborhood cohesion" OR "collective efficacy") | 58 |
| **Life Satisfaction** | (“life satisfaction” OR “satisfaction with life” OR “quality of life” OR “personal satisfaction” OR “life dissatisfaction”) | 89 |
| **Education** | (education OR educational OR achievement OR “educational attendance” OR “educational attainment” OR “educational level” OR “educational background” OR “education failure” OR “level of education” OR “years of education” OR “academic attainment” OR “academic achievement” OR “academic background” OR qualification) | 422 |
| **Economic factors (employment, income, wealth)** | (employment OR employed OR unemployed OR unemployment OR career OR “career mobility” OR job OR jobs OR jobless OR joblessness OR work OR income OR wage OR salary OR pay OR earning OR earnings OR productive OR productivity OR “quality of employment” OR wealth OR “asset accumulation” OR savings OR “assets accumulated” OR poor OR poverty OR savings OR “job satisfaction” OR “socioeconomic status” OR “socio-economic status” OR economic OR stature) | 778 |
| *Note.* The searches were conducted separately for physical and mental health in the Web of Science Core Collection, with key words in the Topic – one for physical health and one for mental health. In addition, separate searches were conducted for each social or economic outcome. Each search included key terms for health (physical or mental), studies, association and one of the outcomes. For instance, the structure of the search for physical health and social capital was: Physical Health AND Studies AND Association and Social Capital. | | |
