# Supplementary material for: The impact of health on economic and social outcomes in the United Kingdom: A scoping literature review
Source: PLoS One. 2018 Dec 31;13(12):e0209659. doi: 10.1371/journal.pone.0209659 (PMC6312330; doi:10.1371/journal.pone.0209659)
Supplement: S1 File — (DOC) [file pone.0209659.s003.doc]

| Table A. | | | | | |
| --- | --- | --- | --- | --- | --- |
| **Reference** | **Study**  **(sample size)** | **Health predictor (age)-> Outcome (age): strength of the association (SE/CI95%)*** | **Associated (Yes/No)** | **Adjusted covariates** | **Quality of evidence (Good/Fair/Poor)** |
| Booth et al. (2014) | ALSPAC (5966) | 1) Obesity (11; female)->lower academic attainment (English mark; 11, 13, 16):  B=-1.35 (-3.17 to 0.48);  -3.71 (-6.38 to -1.04); -0.26 (-0.47 to -0.05) | Yes | Age; birth weight; gestation; age of mother at delivery; oily fish intake during pregnancy; whether mother smoked during pregnancy; pubertal status; ethnicity, maternal education; maternal social class; average daily number of minutes spent in MVPA; depressive symptoms at 11; full-scale IQ at 8 years. | **Good** |
|  |  | 2) Obesity (11; male)->academic attainment (English mark; 11, 13, 16):  B=-2.52 (-4.78 to - 0.26);  -1.59 (-4.76 to 1.58); -0.08 (-0.33 to 0.16) | No |  |  |
| Butland et al. (2010) | NCDS (5020) | 1) Fever/allergic rhinitis (7, 11, 16)->lower likelihood of having a first job with a high risk of asthma based on job title (33):  RRR=0.70; 0.56-0.88) | Yes | Sex, father’s social class at birth, region, school leaving age, further education on leaving school, and for each other. | **Good** |
|  |  | 2) Eczema (7, 11, 16)->likelihood of having a first job with a high risk of asthma based on job title (33): RRR=1.14 (0.90-1.46) | No |  |  |
|  |  | 3) Asthma/wheezy bronchitis (11-16)->higher likelihood of having a first job with a high risk of asthma based on job title (33):  RRR=1.60 (1.17-2.19) | Yes |  |  |
|  |  | 4) Fever/allergic rhinitis (7, 11, 16)->lower likelihood of having a first job with a high risk of asthma based on exposure matrix (33): RRR=0.77 (0.62-0.96) | Yes |  |  |
|  |  | 5) Eczema (7, 11, 16)->likelihood of having a first job with a high risk of asthma based on exposure matrix (33): RRR=1.08 (0.85-1.38) | No |  |  |
|  |  | 6) Asthma/wheezy bronchitis (11-16)-> likelihood of having a first job with a high risk of asthma based on exposure matrix (33): RRR=1.18 (0.85-1.64) | No |  |  |

| Table A. (cont.) | | | | | |
| --- | --- | --- | --- | --- | --- |
| **Reference** | **Study**  **(sample size)** | **Health predictor (age)-> Outcome (age): strength of the association (SE/CI95%)*** | **Associated (Yes/No)** | **Adjusted covariates** | **Quality of evidence (Good/Fair/Poor)** |
| Case & Paxson (2008) | NCDS (4860- 12,449) | 1) Height (7-16; male)->higher average hourly earnings (33/42): B=-0.003 (0.006) to 0.018 (0.007) | Yes | Indicators of mother and father school leaving ages, mother’s father’s social class, low birth weight, and controls for mother’s and father’s heights, and log family income in childhood. | **Fair** |
|  |  | 2) Height (7-16; female)->average hourly earnings (33/42): B=-0.008 (0.008) to 0.010 (0.007) | No |  |  |
| Chin et al. (2011) | NCDS (101 - ?) | 1) Epilepsy (16 or under)->achieved A level/higher education (33): OR=0.69 (0.34–1.37) | No | Cognitive ability, anxiety about peer/adult acceptance at age, peer/adult and adult acceptance at age, maternal interest in education. | **Good** |
|  |  | 2) Epilepsy (16 or under)->ever been in paid employment (33): OR=0.56 (0.17-1.81) | No |  |  |
|  |  | 3) Epilepsy (16 or under)->ever been married (33): OR=0.50 (0.38–0.95) | Yes |  |  |
|  |  | 4) Epilepsy (16 or under)->ever been a parent (33): OR=0.66 (0.42–0.91) | Yes |  |  |
| Conti et al. (2011) | BCS70 (7397) | 1) Higher height and the head circumference (10; male)  ->school attainment (16): estimates not available | No | Accounts for cognitive ability; match on both observables and unobservables where the unobservables are proxied, and account for the errors in the proxies for the unobservables. | **Fair** |
|  |  | 2) Higher height and the head circumference (10; female)  ->higher school attainment (16): estimates not available | Yes |  |  |
|  |  | 3) Higher height and the head circumference (10; male)  ->higher hourly wages (30): estimates not available | Yes |  |  |
|  |  | 4) Higher height and the head circumference (10; female)  ->higher hourly wages (30): estimates not available | Yes |  |  |
|  |  | 5) Higher height and the head circumference (10; male)  ->more likely employed (30): estimates not available | Yes |  |  |
|  |  | 6) Higher height and the head circumference (10; female)  ->more likely employed (30): estimates not available | Yes |  |  |

| Table A. (cont.) | | | | | |
| --- | --- | --- | --- | --- | --- |
| **Reference** | **Study**  **(sample size)** | **Health predictor (age)-> Outcome (age): strength of the association (SE/CI95%)*** | **Associated (Yes/No)** | **Adjusted covariates** | **Quality of evidence (Good/Fair/Poor)** |
| Currie & Hyson (1999) | NCDS (3890-5181) | 1) Low birthweight (female)->less O-level passed (16): B=-0.120 (0.049) to -0.110 (0.043) | Yes | Measures for twins, parent's education, maternal grandfather's SES, single parenthood, family size, birth order, mother's age, and maternal smoking during pregnancy. | **Good** |
|  |  | 2) Low birthweight (male)->less O-level passed (16): B=-0.130 (0.049) to -0.039 (0.046) | Yes |  |  |
|  |  | 3) Low birthweight (female)->unemployed (23/33): B=-0.091 (0.039) to -0.010 (0.040) | Yes |  |  |
|  |  | 4) Low birthweight (male)->unemployed (23/33): B=-0.082 (0.034) to -0.045 (0.035) | Yes |  |  |
|  |  | 5) Low birthweight (female)->salary (23/33): B=-0.035 (0.029) to 0.034 (0.039) | No |  |  |
|  |  | 6) Low birthweight (male)->salary (23/33): B=-0.017 (0.029) to -0.021 (0.045) | No |  |  |
| Griffiths et al. (2006) | ALSPAC (6932-8210) | 1) Obesity (7; female)->being a bully (8):  OR=0.91 (0.58 - 1.43); 1.53 (1.09 to 2.15) | No | Maternal and paternal social class. | **Poor** |
|  |  | 2) Obesity (7; male)->more likely being a bully (8):  OR=1.40 (0.93-2.11); 1.54 (1.12-2.13) | Yes |  |  |
| Griffiths et al. (2011) | MCS (11202) | 1) Obesity (3, male)->conduct problems (5):  B=0.10 (-0.15 to 0.35) | No | Child’s ethnicity; maternal socio-economic status; mother’s highest academic qualification; maternal age at birth; household income; lone-motherhood status; no. of children in the household; maternal treatment for depression or anxiety; maternal weight status. | **Good** |
|  |  | 2) Obesity (3, female)->conduct problems (5):  B=-0.03 (-0.26 to 0.21) | No |  |  |
|  |  | 3) Obesity (3, male)->prosocial behaviour (5):  B=0.03 (-0.25 to 0.31) | No |  |  |
|  |  | 4) Obesity (3, female)->prosocial behaviours (5):  B=0.03 (-0.10 to 0.17) | No |  |  |
|  |  | 5) Obesity (3, male)->more peer problems (5):  B=0.26 (0.01 to 0.52) | Yes |  |  |
|  |  | 6) Obesity (3, female)->peer problems (5):  B=-0.04 (-0.25 to 0.18) | No |  |  |

| Table A. (cont.) | | | | | |
| --- | --- | --- | --- | --- | --- |
| **Reference** | **Study**  **(sample size)** | **Health predictor (age)-> Outcome (age): strength of the association (SE/CI95%)*** | **Associated (Yes/No)** | **Adjusted covariates** | **Quality of evidence (Good/Fair/Poor)** |
| Henderson et al. (2013) | BCS70 (14105) | 1) Mother`s rating of health (16)->receiving Incapacity Benefit/Severe Disablement Allowance (34):  OR=2.7 (1.3–5.3) | Yes | Adult health variables at age 30, maternal rating of participant’s health at age 16, model, depression/ anxiety/headache/stomach ache all measured at age 16. | **Good** |
| Holme et al. (2013) | ALSPAC (11534) | 1) Weight faltering (0)->education (6-11):  OR=1.08 (0.75-1.55) to 1.65 (1.15 to 2.37) | No | Gender; the child’s IQ; birthweight, breastfeeding; maternal age and education; maternal smoking; family size; housing tenure; a home facilities score at 6 months; paternal socioeconomic status at the time of pregnancy; Family Adversity Index (e.g. housing quality, financial difficulties); parenting attitudes at 6 months. | **Fair** |
|  |  | 2) Weight faltering (0)->self-esteem (8):  OR=0.81 (0.65-1.01); 0.96 (0.77-1.20) | No |  |  |
|  |  | 3) Weight faltering (0)->more likely peer relationships (8): OR=1.26 (1.01-1.56) | Yes |  |  |
|  |  | 4) Weight faltering (0)->experience of bullying (8): OR=0.97 (0.67-1.39); 1.17 (0.89-1.53) | No |  |  |
|  |  | 5) Weight faltering (0)->social cognition (7):  OR=0.87 (0.67 to 1.12) | No |  |  |
|  |  | 6) Weight faltering (0)->antisocial activities (8): OR=1.08 (0.67-1.77) | No |  |  |
| Kelly et al. (2016) | MCS (8259) | 1) High Increasing BMI trajectory (3-11)->more peer problems (11): B=0.84 (0.58 to 1.11) | Yes | Mother smoked during pregnancy; child was breastfed; solids introduced before 4 months; gross motor delay; maternal BMI; sugary drinks; fruit consumption; skip breakfast; sports participation; TV viewing; on-regular bedtime; late bedtime. | **Good** |
|  |  | 2) High Increasing BMI trajectory (3- 11)->prosocial behaviour (11): B=0.16 (-0.04 to 0.35) | No |  |  |
|  |  | 3) High Increasing BMI trajectory (3-)->antisocial activities (11): B=0.03 (-0.06 to 0.11) | No |  |  |
| Khandaker et al. (2015) | ALSPAC (5883) | 1) Meningitis (18 months)->worse educational abilities (10): B=0.46 (-0.49 to 1.40) to 1.01 (0.01 to 2.02) | Yes | Age at the time of testing; sex; social class; ethnicity; father’s social class. | **Fair** |
| Kline & Tobias (2008) | BCS70 (4343) | 1) Obesity (5, 10, 16, 26, female)->lower hourly wages (29/30): estimates not available | Yes | Time in current job; labour market experience; education; marital status; union membership; family income at 10 years of age; parental education; parental social class; parental BMI. | **Fair** |
|  |  | 2) Obesity (5, 10, 16, 26, male)->lower hourly wages (29/30): estimates not available | Yes |  |  |

| Table A. (cont.) | | | | | |
| --- | --- | --- | --- | --- | --- |
| **Reference** | **Study**  **(sample size)** | **Health predictor (age)-> Outcome (age): strength of the association (SE/CI95%)*** | **Associated (Yes/No)** | **Adjusted covariates** | **Quality of evidence (Good/Fair/Poor)** |
| Montgomery et al. (1996) | NCDS (2256 male only) | 1) Higher height (7)->more likely employed (22-32): OR=2.41 (1.43-4.04) | Yes | Social class at birth, crowding at age 7, educational attainment, region of residence; parental socioeconomic background, parental education, and parental height. | **Fair** |
|  |  | 2) Externalising problems (11)->more likely unemployed (22-32): OR=2.36 (1.49-3.73) | Yes |  |  |
| Murasko (2007) | BCS70 (4547) | 1) Height (10)->educational attainment (16):  B=0.353 (0.349) | No | Age 10: psychosocial measures; social class; mother’s education; cognitive ability. | **Good** |
|  |  | 2) Weight (10)->educational attainment (16):  B=-0.022 (0.085) | No |  |  |
|  |  | 3) Has 2+ abnormal conditions->educational attainment (16): B=0.086 (0.118) | No |  |  |
| Palloni et al. (2009) | NCDS (3677) | 1) Chronic conditions (7)->lower social class (42):  B=-0.178 (0.091) | Yes | Parental social class; maternal health; low birthweight; cognitive score at age 11; behavioural maladjustment at age 11; educational attainment at age 20. | **Fair** |
|  |  | 2) Chronic conditions (16)->social class (42):  B=-0.011 (0.054) | No |  |  |
|  |  | 3) Chronic conditions (7)->educational attainment (20): B=0.097 (0.288) | No |  |  |
|  |  | 4) Chronic conditions (16)->educational attainment (20): B=-0.011 (0.153) | No |  |  |
|  |  | 5) Birthweight->social class (42): B=0.034 (0.143) | No |  |  |
| Peet et al. (2015) | BCS70 (6533) | 1) Better physical development (height; 2, 5)->higher educational attainment (16+): B=0.103 (0.042-0.164) | Yes | Region fixed effects; mother's and father's years of education; paternal social class; low birthweight; mother's height; number of previous pregnancies; age at childbirth; smoking during pregnancy.  Additional analyses: parent’s marital status; number of antenatal visits to healthcare provider; gestational length, mother’s employment status; delivery complications. | **Fair** |

| Table A. (cont.) | | | | | |
| --- | --- | --- | --- | --- | --- |
| **Reference** | **Study**  **(sample size)** | **Health predictor (age)-> Outcome (age): strength of the association (SE/CI95%)*** | **Associated (Yes/No)** | **Adjusted covariates** | **Quality of evidence (Good/Fair/Poor)** |
| Persico et al. (2004) | NCDS (1772; men only) | 1) Higher height (7, 11, 16)->higher earnings (33):  B=0.020 (0.0065) | Yes | Adult height, years of completed schooling; ever married; divorced or separated; family background; mother’s/father`s years of schooling; mother’s/father`s occupational status; number of siblings. | **Fair** |
| Rahi et al. (2006) | NCDS (5161) | 1) Amblyopia (16 or under)->education (up to 16 ):  B=-0.86 (-2.99 to 1.27) to 0.09 (-0.03 to 0.20) | No | Social class; sex; age at testing; family size; ever having strabismus; treatment for amblyopia; marital status; and other exploratory variables when appropriate. | **Fair** |
|  |  | 2) Amblyopia (16 or under)->participation in sport/outdoor games (11-23): OR=0.76 (0.46-1.25) to 1.07 (0.65-1.75) | No |  |  |
|  |  | 3) Amblyopia (16 or under)->social activities (16-23): B=0.86 (0.49-1.53) | No |  |  |
|  |  | 4) Amblyopia (16 or under)->education (33):  OR=0.99 (0.61-1.61) | No |  |  |
|  |  | 5) Amblyopia (16 or under; male)->employment history (33): OR=0.51 (0.22-1.17) | No |  |  |
|  |  | 6) Amblyopia (16 or under; female)->employment history (33): OR=0.71 (0.31-1.64) | No |  |  |
| Sargent & Blanchflower (1994) | NCDS (12537) | 1) Obesity (16; male)->hourly earnings (23):  B=-3.7 (-4.9 to 4.2) | No | Social class; IQ; unemployment rate; type of industry; marital status; highest educational qualifications; plant size; part-time dummy; union membership; ethnicity. | **Fair** |
|  |  | 2) Obesity (16; female)->lower hourly earnings (23):  B=-5.3 (-8.3 to -2.3) | Yes |  |  |
|  |  | 3) Obesity (16, 23; male)->hourly earnings (23):  B=-2.8 (1.0) | No |  |  |
|  |  | 4) Obesity (16, 23; female)->lower hourly earnings (23):  B=-6.4 (2.1) | Yes |  |  |
|  |  | 5) Obesity (16; male)->employment (23):  estimates not available | No |  |  |
|  |  | 6) Obesity (16; female)->employment (23):  estimates not available | No |  |  |

| Table A. (cont.) | | | | | |
| --- | --- | --- | --- | --- | --- |
| **Reference** | **Study**  **(sample size)** | **Health predictor (age)-> Outcome (age): strength of the association (SE/CI95%)*** | **Associated (Yes/No)** | **Adjusted covariates** | **Quality of evidence (Good/Fair/Poor)** |
| Schick & Steckel (2015) | NCDS (6521) | 1) Higher height (7; male)->better reading scores (11):  B=0.130 (0.018) | Yes | Ethnicity; region; medical exam date; household income; father’s socioeconomic group; mother’s and father’s height; education; involvement in child’s education; cognitive and personality measures. | **Good** |
|  |  | 2) Higher height (7; female)->better reading scores (11):  B=0.116 (0.016) | Yes |  |  |
|  |  | 3) Higher height (7; male)->better math scores (11):  B=0.106 (0.017) | Yes |  |  |
|  |  | 4) Higher height (7; female)->better math scores (11):  B=0.122 (0.016) | Yes |  |  |
| Sibbald et al. (1992) | NCDS (8379) | 1) Asthma (7, 11, 16, 23)->more likely unemployed (23):  OR=1.32 (1.09-1.61); 1.54 (1.27-1.85) | Yes | Sex; region of birth; maximum educational qualification; father's social class. | **Fair** |
|  |  | 2) Asthma (7, 11, 16, 23; male)->number of months  unemployed before first job (23):  B=-0.0 (-0.3 to 0.2); 0.1 (-0.3 to 0.3) | No |  |  |
|  |  | 3) Asthma (7, 11, 16, 23; male)->changed jobs more frequently (23): B=0.19 (-0.10 to 0.48); 0.23 (0.11 to 0.35) | Yes |  |  |
|  |  | 4) Asthma (7, 11, 16, 23; male)->number of periods unemployed (23): B=0.2 (-0.1 to 0.5); 0.1 (-1.8 to 0.2) | No |  |  |
|  |  | 5) Asthma (7, 11, 16, 23; male)->lower social group (23): OR=0.88 (0.69-1.12); 0.91 (0.83-0.99) | Yes |  |  |
|  |  | 6) Asthma (7, 11, 16, 23; female)->number of months  unemployed before first job (23):  B=0.0 (-0.2 to 0.2); 0.1 (-0.0 to 0.3) | No |  |  |
|  |  | 7) Asthma (7, 11, 16, 23; female)->changed jobs more frequently (23): B=0.30 (0.60 to 0.54); 0.03 (-0.09 to 0.15) | Yes |  |  |
|  |  | 8) Asthma (7, 11, 16, 23; female)->number of periods unemployed (23): B=0.1 (-0.1 to 0.3); 0.0 (-0.1 to 0.1) | No |  |  |
|  |  | 9) Asthma (7, 11, 16, 23; female)->a lower social group (23): OR=0.92 (0.79-1.07); 0.94 (0.88-1.00) | Yes |  |  |

| Table A. (cont.) | | | | | |
| --- | --- | --- | --- | --- | --- |
| **Reference** | **Study**  **(sample size)** | **Health predictor (age)-> Outcome (age): strength of the association (SE/CI95%)*** | **Associated (Yes/No)** | **Adjusted covariates** | **Quality of evidence (Good/Fair/Poor)** |
| Teyhan et al. (2015) | ALSPAC (6626) | 1) Persistent symptoms of rash (1-8)->more likely to be bullied (8): OR=1.29 (1.02-1.63) | Yes | Child age and sex, maternal age, maternal education, financial difficulties, housing tenure, wheeze (in rash model) or rash (in wheeze model). | **Good** |
|  |  | 2) Persistent symptoms of rash (1-8)->being happy at school (8): OR=1.04 (0.88-1.22) | No |  |  |
|  |  | 3) Persistent symptoms of rash (1-8)->feeling left out (8): OR=1.01 (0.80-1.28) | No |  |  |
|  |  | 4) Persistent symptoms of wheeze (1-8)->feeling left out (8): OR=1.42 (1.10-1.84) | Yes |  |  |
|  |  | 5) Persistent symptoms of wheeze (1-8)->being bullied (8): OR=1.11 (0.85-1.45) | No |  |  |
|  |  | 6) Persistent symptoms of wheeze (1-8)->being happy at school (8): OR=1.14 (0.95-1.36) | No |  |  |
| Scholder et al. (2012) | ALSPAC (3001) | 1) OLS: obesity/body fat mass (9, 11)->better educational outcomes (English, math, science) (11, 14): B=-0.037 (0.015); -0.025 (0.015) | Yes | Birthweight; number of older and younger siblings under 18; age in months; family income; mother’s educational level; mother’s parents’ educational level; raised by natural father; social class; maternal age at birth; parents’ employment status and IMD; mother’s health and behaviour (e.g. mother’s smoking and drinking during pregnancy, breastfeeding). | **Good** |
|  |  | 2) Fixed effects: obesity/body fat mass (9, 11)->better educational outcomes (English, math, science) (11, 14): B=-0.030 (0.017); -0.030 (0.017) | Yes |  |  |
|  |  | 3) Instrumental variable: mother’s prepregnancy BMI  ->better educational outcomes (English, math, science) (11, 14): B=-0.119 (0.046) | Yes |  |  |
|  |  | 4) Instrumental variable: child’s lagged adiposity-> better educational outcomes (English, math, science) (11, 14): B=-0.031 (0.017) | Yes |  |  |
|  |  | 5) Instrumental variable: genetic markers-> educational outcomes (English, math, science) (11, 14): B=-0.039 (0.122) | No |  |  |

| Table A. (cont.) | | | | | |
| --- | --- | --- | --- | --- | --- |
| **Reference** | **Study**  **(sample size)** | **Health predictor (age)-> Outcome (age): strength of the association (SE/CI95%)*** | **Associated (Yes/No)** | **Adjusted covariates** | **Quality of evidence (Good/Fair/Poor)** |
| Viner & Cole (2005) | BCS70 (8490-12160) | 1) Obesity (10; male)->low social class (30): OR=1.4 (0.8-2.5) | No | Maternal education, social class in childhood and adulthood, maternal and paternal BMI, and height at 10 and 30 years. | **Good** |
|  |  | 2) Obesity (10; male)->left school aged ≤16 (30): OR=0.9 (0.5-1.5) | No |  |  |
|  |  | 3) Obesity (10; male)->left school with no qualifications (30):  OR=0.8 (0.4-1.4) | No |  |  |
|  |  | 4) Obesity (10; male)->never married/has no partner (30):  OR=0.9 (0.6-1.4); 1.1 (0.7-1.7) | No |  |  |
|  |  | 5) Obesity (10; male)->unemployment (30): OR=0.8 (0.2-3.2) | No |  |  |
|  |  | 6) Obesity (10; female)->low social class (30): OR=0.9 (0.5-1.6) | No |  |  |
|  |  | 7) Obesity (10; female)->left school aged ≤16 (30): OR=1.2 (0.7-2.0) | No |  |  |
|  |  | 8) Obesity (10; female)->left school with no qualifications (30): OR=1.0 (0.6-1.7) | No |  |  |
|  |  | 9) Obesity (10; female)->never married/has no partner (30):  OR=0.7 (0.4-1.1); 1.2 (0.8-2.1) | No |  |  |
|  |  | 10) Obesity (10; female)->unemployment (30): OR=0.6 (0.1-5.4) | No |  |  |
|  |  | 11) Obesity (10 and 30; male)->low social class (30): OR=1.2 (0.7-0.2) | No |  |  |
|  |  | 12) Obesity (10 and 30; male)->left school aged ≤16 (30):  OR=1.0 (0.6-1.6) | No |  |  |
|  |  | 13) Obesity (10 and 30; male)->left school with no qualifications (30): OR=1.2 (0.7-1.8) | No |  |  |
|  |  | 14) Obesity (10 and 30; male)->never married/has no partner (30): OR=0.8 (0.5-1.2); 0.8 (0.5-1.3) | No |  |  |
|  |  | 15) Obesity (10 and 30; male)->unemployment (30): OR=1.4 (0.5-3.7) | No |  |  |
|  |  | 16) Obesity (10 and 30; female)->low social class (30):  OR=1.3 (0.7-2.1) | No |  |  |
|  |  | 17) Obesity (10 and 30; female)->left school aged ≤16 (30):  OR=0.9 (0.5-1.4) | No |  |  |
|  |  | 18) Obesity (10 and 30; female)->left school with no qualifications (30): OR=0.7 (0.4-1.3) | No |  |  |
|  |  | 19) Obesity (10 and 30; female)->never married/has no partner (30): OR=1.3 (0.8-2.1); 2.0 (1.3-3.3) | Yes |  |  |
|  |  | 20) Obesity (10 and 30; female)->unemployment (30):  OR=0.8 (0.1-5.8) | No |  |  |

| Table B. | | | | | |
| --- | --- | --- | --- | --- | --- |
| **Reference** | **Study**  **(sample size)** | **Health predictor (age)-> Outcome (age): strength of the association (SE/CI95%)*** | **Associated (Yes/No)** | **Adjusted covariates** | **Quality of evidence (Good/Fair/Poor)** |
| Asher et al. (2013) | ALSPAC (3077) | 1) Psychotic symptoms (13)->social functioning (peer problems) (13): OR=1.12 (0.43-2.91) | No | Social functioning (SDQ peer problems score) at age 11; mother's social class; housing tenure; gender; maternal education; maternal smoking during pregnancy; mother's marital status; IQ; family psychiatric history; bullying age 8; depression age 12; conduct problems, hyperactivity and emotional symptoms age 11. | **Fair** |
| Byford et al. (2013) | NSHD (1110) | 1) Conduct and emotional problems (13, 15)  ->coercive discipline (19-29):  B=0.17 (-0.02 to 0.37) to 0.17 (-0.02 to 0.37) | No | Parent and offspring sex, adolescent cognition, household social class social class and educational attainment. | **Fair** |
|  |  | 2) Conduct and emotional problems (13, 15)  ->intellectual environment (19-29):  B=0.20 (-0.11 to 0.51); 0.08 (-0.16 to 0.33) | No |  |  |
|  |  | 3) Conduct and emotional problems (13, 15)  ->cognitive stimulation (19-29):  B=0.09 (-0.12 to 0.30); -0.06 (-0.22 to 0.12) | No |  |  |
|  |  | 4) Conduct and emotional problems (13, 15)  ->parental interest (19-29):  B=-0.13 (-0.33 to 0.07); 0.11 (-0.15 to 0.37) | No |  |  |
|  |  | 5) Conduct and emotional problems (13, 15)  ->parental aspiration (19-29):  B=-0.07 (-0.42 to 0.27); 0.01 (-0.42 to 0.43) | No |  |  |
| Caes et al. (2014) | ALSPAC (856) | 1) Pain-related anxiety (17; female)->greater social impairment (17): B=0.228 (p<0.001) | Yes | Parental social class, pain intensity. | **Poor** |
|  |  | 2) Pain-related anxiety (17; male)->social impairment (17): B=0.047 (p>0.05) | No |  |  |
| Williams et al. (2011) | ALSPAC (4512) | 1) Visual difficulties (13)->worse reading abilities (13-14): B=0.02 (0.01 to 0.03) | Yes | Age at testing; gender; maternal education; highest maternal/paternal social class; ICD10 diagnosis; visual problems, born at less than 37 weeks gestation; admitted to a Special Care Baby Unit in first month; low birthweight; total IQ. | **Good** |
|  |  | 2) Visual difficulties (13)->worse mathematics abilities (13-14): B=0.02 (0.01 to 0.03) | Yes |  |  |

| Table B. (cont.) | | | | | |
| --- | --- | --- | --- | --- | --- |
| **Reference** | **Study**  **(sample size)** | **Health predictor (age)-> Outcome (age): strength of the association (SE/CI95%)*** | **Associated (Yes/No)** | **Adjusted covariates** | **Quality of evidence (Good/Fair/Poor)** |
| Colman et al. (2009) | NSHD (3652) | 1) Externalising problems (13, 15)->unemployed at least once (36, 43, 53): OR=1.2 (0.7-2.2) | No | Sex; father’s social class; cognitive ability; depression/anxiety in adolescence. | **Fair** |
|  |  | 2) Externalising problems (13, 15)->early parenthood (under 20) more likely: OR=2.4 (1.3-4.4) | Yes |  |  |
|  |  | 3) Externalising problems (13, 15)->one or more divorce (36, 43, 53): OR=1.7 (1.2-2.5) | Yes |  |  |
|  |  | 4) Externalising problems (13, 15)->unhappy with family life (36, 43): OR=1.6 (1.1-2.2) | Yes |  |  |
|  |  | 5) Externalising problems (13, 15)->problems in relationships (36, 43, 53): OR=1.8 (1.3-2.5) | Yes |  |  |
|  |  | 6) Externalising problems (13, 15)->no educational qualifications (26): OR=4.0 (2.9-5.5) | Yes |  |  |
|  |  | 7) Externalising problems (13, 15)->manual social class (36, 43, 53): OR=2.0 (1.5-2.8) | Yes |  |  |
|  |  | 8) Externalising problems (13, 15)->financial difficulties (36, 43, 53): OR=2.1 (1.4-3.2) | Yes |  |  |
|  |  | 9) Externalising problems (13, 15)->global life adversity more likely (53): OR=2.9 (2.1-4.0) | Yes |  |  |
| Egan et al. (2015) | NCDS (8985)  LSYPE (10232) | 1) LSYPE: Psychological distress (14)->unemployment (16, 21):  B=0.287 (0.087) | Yes | SES; derived from their occupation; childhood environmental factors; demographic measures; childhood physical health; adult distress. | **Good** |
|  |  | 2) NCDS: Psychological distress (7, 11)->unemployment (16, 23):  B=0.568 (0.119) | Yes |  |  |
| Farmer (1995) | NCDS (2996; male only) | 1) Externalising problems (11)->school-leaving age (23): B=-0.55 (p>0.05) | No | Father's social class and academic ability. | **Good** |
|  |  | 2) Externalising problems (16)->school-leaving age (23): B=-3.52 (p<0.05) | Yes |  |  |
|  |  | 3) Externalising problems (11)->level of qualification at labour force entry (23): B=-0.13 (p>0.05) | No |  |  |
|  |  | 4) Externalising problems (16)->level of qualification at labour force entry (23): B=-3.52 (p<0.05) | Yes |  |  |
|  |  | 5) Externalising problems (11)->social class (23): B=0.04 (p>0.05) | No |  |  |

| Table B. (cont.) | | | | | |
| --- | --- | --- | --- | --- | --- |
| **Reference** | **Study**  **(sample size)** | **Health predictor (age)-> Outcome (age): strength of the association (SE/CI95%)*** | **Associated (Yes/No)** | **Adjusted covariates** | **Quality of evidence (Good/Fair/Poor)** |
| Farmer (1995) continued | As above | 6) Externalising problems (16)->social class (23):  B=-0.06 (p>0.05) | No | As above. | As above |
|  |  | 7) Persistent externalising problems (7-16)->school-leaving age (23): B=-6.79 (p<0.0001) | Yes |  |  |
|  |  | 8) Persistent externalising problems (7-16)->level of qualification at labour force entry (23): B=-1.16 (p<0.0001) | Yes |  |  |
|  |  | 9) Persistent externalising problems (7-16)->social class (23): B=-0.41 (p<0.0001) | Yes |  |  |
| Flouri (2007) | BCS70 (6522) | 1) Hyperactivity(5)->worse educational attainment (26):  B=-0.08 (0.01) | Yes | Sex of child, socio-economic disadvantage at age five, mother’s age at birth (teenager or not), parental family structure; general ability (age 5). | **Fair** |
| Johnston et al. (2013) | BCS70 (8194) | 1) Psychological distress (10)->having a degree-level qualification (by age 30): B=-0.023 (0.005) | Yes | Maternal mental health; childhood health at age 0 and age 5; family characteristics at age 5; childhood test scores at age 5; childhood mental health at age 5; physical healt at age 30; degree level qualification (where relevant); maternal education; parental social class; no father in household; vocabulary and ability tests. | **Good** |
|  |  | 2) Psychological distress (10)->household income (30, 34): B=0.003 (0.008) | No |  |  |

| Table B. (cont.) | | | | | |
| --- | --- | --- | --- | --- | --- |
| **Reference** | **Study**  **(sample size)** | **Health predictor (age)-> Outcome (age): strength of the association (SE/CI95%)*** | **Associated (Yes/No)** | **Adjusted covariates** | **Quality of evidence (Good/Fair/Poor)** |
| Mars et al. (2014) | ALSPAC (4799) | 1) Suicidal self-harm (16)->low educational attainment (16, 19): OR=2.08 (1.47-2.95) | Yes | Sex; depressive symptoms; IQ at age 8; socioeconomic position (includes maternal education and parental social class). | **Fair** |
|  |  | 2) Non-suicidal self-harm (16)->low educational attainment (16, 19): OR=1.15 (0.81-1.63) | No |  |  |
|  |  | 3) Suicidal self-harm->more likely NEET (19): OR=1.96 (1.16-3.31) | Yes |  |  |
|  |  | 4) Non-suicidal self-harm (16)->NEET (19): OR=1.41 (0.86-2.33) | No |  |  |
| Maughan & Taylor (2001) | NCDS (5773) | 1) Psychological distress (16; male)->less likely to form partnership (up to 33): HR=0.66 (0.55-0.79); 0.69 (0.52-0.91) | Yes | Childhood social class; education; parental divorce; age at first partnership (when relevant); having a child (when relevant); type of partnership (when relevant). | **Good** |
|  |  | 2) Psychological distress (16; female)->later age of partnership formation (up to 33): p>0.05; estimates not available | No |  |  |
|  |  | 3) Psychological distress (16; male)->breakdown of first partnership (up to 33): p>0.05; estimates not available | No |  |  |
|  |  | 4) Psychological distress (16; female)->breakdown of first partnership (up to 33): p>0.05; estimates not available | No |  |  |
| Nishida et al. (2016) | NSHD (1561) | 1) Emotional problems (13/15)->lower life satisfaction (60-64):  B=-0.052 (p=0.034) | Yes | Emotional problems/ conduct problems/ self-regulation (where appropriate); gender; childhood cognitive ability at age 8; father’s occupational social class; educational attainment by age 26; GHQ-28 total score at age 60–64. | **Fair** |
|  |  | 2) Conduct problems (13/15)->life satisfaction (60-64):  B=-0.003 (p=0.9) | No |  |  |
| Sayal et al. (2015) | ALSPAC (11640) | 1) ADHD (7; male)->failure to achieve 5 good GCSEs (16):  OR=2.11 (1.05-4.21) | Yes | Mutually adjusted for inattention, hyperactivity/ impulsivity, and oppositional/defiant behaviours, and for corresponding diagnosis and interaction; child IQ; parental education and social class; early maternal depression. | **Good** |
|  |  | 2) ADHD (7; female)->failure to achieve 5 good GCSEs (16): OR=1.80 (0.96-3.39) | No |  |  |
|  |  | 3) DBD (7; male)->failure to achieve 5 good GCSEs (16):  OR=1.83 (1.22-2.76) | Yes |  |  |
|  |  | 4) DBD (7; female)->failure to achieve 5 good GCSEs (16):  OR=1.77 (0.88-3.55) | No |  |  |

| Table B. (cont.) | | | | | | |
| --- | --- | --- | --- | --- | --- | --- |
| **Reference** | **Study**  **(sample size)** | | **Health predictor (age)-> Outcome (age): strength of the association (SE/CI95%)*** | **Associated (Yes/No)** | **Adjusted covariates** | **Quality of evidence (Good/Fair/Poor)** |
| Stansfeld et al. (2007) | NCDS (8243) | | 1) Internalising problems (16)->low job demands (45):  RR=0.80 (0.71-0.91) | Yes | Sex; housing tenure at age 7; marital status at 45; educational attainment at 33; physical ill health at 42; mental health diagnosis at 45. | **Good** |
|  |  |  | 2) Internalising problems (16)->low decision latitude (45):  RR=1.51 (1.31-1.74) | Yes |  |  |
|  |  |  | 3) Internalising problems (16)->low work social support (45):  RR=1.20 (1.05-1.38) | Yes |  |  |
|  |  |  | 4) Internalising problems (16)->high job strain (45): OR=1.06 (0.93-1.22) | No |  |  |
|  |  |  | 5) Internalising problems (16)->job insecurity (45): OR=1.08 (0.93-1.26) | No |  |  |
|  |  |  | 6) Internalising problems (16)->lower socioeconomic status (unskilled manual workers; 42): OR=2.71 (1.96-3.77) | Yes |  |  |
| Washbrook et al. (2013) | ALSPAC (11640) | 1) Hyperactivity/inattention (3; male)->five good GCSEs (16):  OR=1.33 (1.09-1.62) | | Yes | Mutually adjusted for hyperactivity/inattention and conduct problems; parental education; social class; early maternal depression; child IQ. | **Good** |
|  |  | 2) Hyperactivity/inattention (3; female)->five good GCSEs (16):  OR=1.24 (0.98-1.56) | | No |  |  |
|  |  | 3) Conduct problems (3; male)->five good GCSEs (16):  OR=1.40 (1.14-1.72) | | Yes |  |  |
|  |  | 4) Conduct problems (3; female)->five good GCSEs (16):  OR=1.32 (1.01-1.73) | | Yes |  |  |

| Table C. | | | | | |
| --- | --- | --- | --- | --- | --- |
| **Reference** | **Study**  **(sample size)** | **Health predictor (age)-> Outcome (age): strength of the association (SE/CI95%)*** | **Associated (Yes/No)** | **Adjusted covariates** | **Quality of evidence (Good/Fair/Poor)** |
| Case et al. (2005) | NCDS (5439-14325) | 1) Chronic conditions (7, 16)->lower educational attainment (total O levels; 16): B=-0.306 (0.057); -0.210 (0.046) | Yes | Maternal smoking; parental education; family income; parental social class; cohort member’s education, and his SES and health at ages 23 and 33 (where relevant). | **Fair** |
|  |  | 2) Low birthweight->lower educational attainment  (total O levels; 16): B=-0.457 (0.091) | Yes |  |  |
|  |  | 3) Systems conditions (7, 16)->lower educational attainment (total O levels; 16): B=-0.289 (0.110); -0.098 (0.089) | Yes |  |  |
|  |  | 4) Physical impairments (7, 16)->educational attainment (total O levels; 16): B=-0.007 (0.125); -0.039 (0.092) | No |  |  |
|  |  | 5) Mental/emotional conditions (7, 16)->lower educational attainment (total O levels; 16): B=-0.601 (0.128); -0.637 (0.123) | Yes |  |  |
|  |  | 5) Low birthweight (male)->unemployment (33, 42):  B=-0.037 (0.021); -0.023 (0.020) | Yes |  |  |
| Chatzitheo-chari et al. (2016) | MCS (7342)  LSYPE  (12144) | 1) MCS: Long-standing limiting illness (3, 5, 7)->being bullied (7): B=0.39 (0.15) | Yes | Employment status; parental closeness to child; discipline measures used; child height and weight; maternal depression; prior cognitive ability; arguments with parents; parental disability; prior educational attainment; complex survey design; nonresponse. | **Fair** |
|  |  | 2) LSYPE: Long-standing limiting illness (13)->being physically bullied (15): B=0.34 (0.214) | No |  |  |
|  |  | 3) LSYPE: Long-standing limiting illness (13)->being relationally bullied (15): B=0.44 (0.162) | Yes |  |  |
| Frijters et al. (2014) | NCDS (4400) | 1) Low birthweight->lower life satisfaction (33-50):  B=-0.215 (t=-2.82) | Yes | Age 7: test scores for arithmetic, copying design, draw-a-man and reading, chronic health conditions, an index of behavioural problems and the British Social Maladjustment Guide score; updated measures of father’s occupational class and dummy indicators for health;  Age 11/16: measures of health, cognitive and non-cognitive attributes, number of siblings, whether or not the biological father is in the household and  father’s occupational status. | **Good** |
|  |  | 2) Number of chronic health conditions (7)->life satisfaction (33-50): B=0.008 (t=0.38) | No |  |  |
|  |  | 3) Any physical handicap/disabling condition (7-11)->life satisfaction (33-50): B=-0.074 (t=-1.10) to -0.043 (t=-0.47) | No |  |  |
|  |  | 4) Behavioural-emotional problems (7-16)->lower life satisfaction (33-50): B=-1.302 (t=-7.32) to 0.119 (t=0.91) | Yes |  |  |
|  |  | 5) Height (11-16)->life satisfaction (33-50):  B=0.397 (t=1.07); 0.517 (t=1.22) | No |  |  |
|  |  | 6) Weight (11-16)->life satisfaction (33-50):  B=-0.005 (t=-1.82); -0.009 (t=-2.13) | No |  |  |

| Table C. (cont.) | | | | | |
| --- | --- | --- | --- | --- | --- |
| **Reference** | **Study**  **(sample size)** | **Health predictor (age)-> Outcome (age): strength of the association (SE/CI95%)*** | **Associated (Yes/No)** | **Adjusted covariates** | **Quality of evidence (Good/Fair/Poor)** |
| Goodman et al. (2011) | NCDS (6113- 11515) | 1) Psychological distress (up to 16)->lower family income (23):  B=-0.195 (p<0.01) | Yes | Family background measures (e.g. education of both parents; occupation status of father); family difficulties during childhood (e.g. financial; physical illness or disability; divorce, separation). | **Good** |
|  |  | 2) Psychological distress (up to 16)->lower family income (33):  B=-0.232 (p<0.01) | Yes |  |  |
|  |  | 3) Psychological distress (up to 16)->lower family income (42):  B=-0.268 (p<0.01) | Yes |  |  |
|  |  | 4) Psychological distress (up to 16)->lower family income (50):  B=-0.301 (p<0.01) | Yes |  |  |
|  |  | 5) Psychological distress (up to 16)->unemployment (23):  B=-0.116 (p<0.01) | Yes |  |  |
|  |  | 6) Psychological distress (up to 16)->unemployment (33):  B=-0.103 (p<0.01) | Yes |  |  |
|  |  | 7) Psychological distress (up to 16)->unemployment (42):  B=-0.107 (p<0.01) | Yes |  |  |
|  |  | 8) Psychological distress (up to 16)->unemployment (50):  B=-0.107 (p<0.01) | Yes |  |  |
|  |  | 9) Psychological distress (up to 16)->likelihood of being married/cohabiting (23): B=-0.027 (p>0.05) | No |  |  |
|  |  | 10) Psychological distress (up to 16)->lower likelihood of being married/cohabiting (33): B=-0.119 (p<0.01) | Yes |  |  |
|  |  | 11) Psychological distress (up to 16)->lower likelihood of being married/cohabiting (42): B=-0.114 (p<0.01) | Yes |  |  |
|  |  | 12) Psychological distress (up to 16)->lower likelihood of being married/cohabiting (50): B=-0.063 (p<0.01) | Yes |  |  |
|  |  | 13) Major physical problems (up to 16)->lower family income (23):  B=-0.067 (p<0.01) | Yes |  |  |
|  |  | 14) Major physical problems (up to 16)->lower family income (33):  B=-0.099 (p<0.01) | Yes |  |  |
|  |  | 15) Major physical problems (up to 16)->lower family income (42):  B=-0.002 (p>0.05) | No |  |  |
|  |  | 16) Major physical problems (up to 16)->lower family income (50):  B=-0.086 (p<0.01) | Yes |  |  |

| Table C. (cont.) | | | | | |
| --- | --- | --- | --- | --- | --- |
| **Reference** | **Study**  **(sample size)** | **Health predictor (age)-> Outcome (age): strength of the association (SE/CI95%)*** | **Associated (Yes/No)** | **Adjusted covariates** | **Quality of evidence (Good/Fair/Poor)** |
| Goodman et al. (2011) continued | See above | 17) Low birth weight->lower family income (23): B=-0.075 (p<0.01) | Yes | See above. | See above |
|  |  | 18) Low birth weight->lower family income (33): B=-0.025 (p>0.05) | No |  |  |
|  |  | 19) Low birth weight->lower family income (42): B=-0.058 (p<0.01) | Yes |  |  |
|  |  | 20) Low birth weight->lower family income (50): B=-0.131 (p<0.01) | Yes |  |  |
|  |  | 21) Psychological distress (up to 16)->downward intergenerational social mobility (42): B=0.120 (p<0.01) | Yes |  |  |
|  |  | 22) Major physical problems (up to 16)->intergenerational social mobility (42): B=-0.003 (p>0.05) | No |  |  |
|  |  | 23) Low birth weight->intergenerational social mobility (42):  B=0.023 (p>0.05) | No |  |  |
|  |  | 24) Psychological distress (up to 16)->downward intragenerational social mobility (23-42): B=0.099 (p<0.01) | Yes |  |  |
|  |  | 25) Major physical problems (up to 16)->intragenerational social mobility (23-42): B=0.009 (p>0.05) | No |  |  |
|  |  | 26) Low birth weight->intragenerational social mobility (23-42):  B=-0.004 (p>0.05) | No |  |  |
|  |  | 27) Psychological distress (up to 16)->employment stability (23-42): B=-0.428 (p<0.01) | Yes |  |  |
|  |  | 28) Major physical problems (up to 16)->employment stability (23-42): B=0.014 (p>0.05) | No |  |  |
|  |  | 29) Low birth weight->employment stability (23-42):  B=-0.089 (p>0.05) | No |  |  |
| Gregg & Machin (2000) | NCDS (6267) | 1) School absence due to sickness (15/16)->continued education (16): B=-0.435 (0.074) | Yes | Ever having been in care, parental education, family structure, parental financial situation. | **Fair** |
|  |  | 2) Externalising problems (7)->continued education (16):  B=-0.357 (0.85) | Yes |  |  |

| Table C. (cont.) | | | | | |
| --- | --- | --- | --- | --- | --- |
| **Reference** | **Study**  **(sample size)** | **Health predictor (age)-> Outcome (age): strength of the association (SE/CI95%)*** | **Associated (Yes/No)** | **Adjusted covariates** | **Quality of evidence (Good/Fair/Poor)** |
| Jackson (2015) | NCDS (9252) | 1) Low birth weight->persistent and accumulating lower academic achievement trajectory in math (7, 11, 16): B=-0.304 (0.05) | Yes | Sex; region; father’s social/occupational class; maternal grandfather’s social class; children`s access to basic resources; mothers’ paid work outside of the home; marital status; number of children in the household; breastfeeding; maternal smoking; birthweight. | **Good** |
|  |  | 2) Poor health/chronic/systemic condition (7)->persistent and accumulating lower academic achievement trajectory in math (7, 11, 16): B=0.021 (0.00) | Yes |  |  |
|  |  | 3) Low birth weight->persistent and accumulating academic achievement trajectory in reading (7, 11, 16): B=0.003 (0.005) | No |  |  |
|  |  | 4) Poor health/chronic/systemic condition (7)->persistent and accumulating academic lower achievement trajectory in reading  (7, 11, 16): B=0.029 (0.005) | Yes |  |  |
|  |  | 5) Persistent and accumulating poor health/chronic/systemic condition (7, 11, 16)->persistent and accumulating lower academic achievement trajectory in math (7, 11, 16): B=-0.154 (0.06) | Yes |  |  |
|  |  | 6) Persistent and accumulating poor health/ chronic/systemic condition (7, 11, 16)->persistent and accumulating lower academic achievement trajectory in reading (7, 11, 16): B=-0.185 (0.05) | Yes |  |  |
| Jackson (2010) | NCDS (9534) | 1) Low birth weight->low NVQ attainment (23): B=-0.281 (3.13) | Yes | Age 7 educational performance; age 11 performance; educational tracking; expectations; age 16/18 performance; and smoking in earlier adulthood. | **Good** |
|  |  | 2) Low birth weight->low NVQ attainment (33): B=-0.271 (3.13) | Yes |  |  |
|  |  | 3) Low birth weight->low NVQ attainment (42): B=-0.292 (3.17) | Yes |  |  |
|  |  | 4) Health problems (7, 11, 16)->low NVQ attainment (23):  B=-1.647 (5.60) | Yes |  |  |
|  |  | 5) Health problems (7, 11, 16)->low NVQ attainment (33):  B=-1.118 (3.83) | Yes |  |  |
|  |  | 6) Health problems (7, 11, 16)->low NVQ attainment (42):  B=-1.261 (4.33) | Yes |  |  |

| Table C. (cont.) | | | | | |
| --- | --- | --- | --- | --- | --- |
| **Reference** | **Study**  **(sample size)** | **Health predictor (age)-> Outcome (age): strength of the association (SE/CI95%)*** | **Associated (Yes/No)** | **Adjusted covariates** | **Quality of evidence (Good/Fair/Poor)** |
| Lee & Jackson (2015) | NCDS (4644) | 1) Health deteriorating then improving (0, 7, 11, 16, 23, 33, 42)->occupational attainment (NVQ; 46):  B=0.09 (0.19) | No | Academic achievement during childhood; employment status during adulthood; parental education; family income; parental social class; mother`s marital status at birth; mother`s age at birth; maternal grandfather’s social class; mother’s smoking status; breastfed as infant; child’s post-school expectations; average number of O-levels. | **Good** |
|  |  | 2) Unhealthy only during adolescence (0, 7, 11, 16, 23, 33, 42)->occupational attainment (NVQ; 46):  B=-0.19 (0.12) | No |  |  |
|  |  | 3) Continuously deteriorating (0, 7, 11, 16, 23, 33, 42)  ->lower occupational attainment (NVQ; 46):  B=-0.22 (0.11) | Yes |  |  |
| Knapp et al. (2011) | BCS70 (8323- 11182) | 1) Childhood antisocial conduct (10; male)  ->unemployment (30): B=-0.089 (p<0.05) | Yes | All at age 10: cognitive skills; motivation; home-life situation; number of children living in household; attended independent sector (privately paid for) school; staff-pupil ratio at school; residence in relatively disadvantaged neighbourhood; parental education; parental hours of work; parental health; family income. | **Good** |
|  |  | 2) Childhood antisocial conduct (10; female)  ->employment (30): B=-0.031 (p>0.05) | No |  |  |
|  |  | 3) Childhood antisocial conduct (10; male)  ->higher earnings (30): B=0.046 (p<0.001) | Yes |  |  |
|  |  | 4) Childhood antisocial conduct (10; female)  ->earnings (30): B=0.022 (p>0.05) | No |  |  |
|  |  | 5) Childhood antisocial conduct (10; male)  ->occupational status (30): B=0.041 (p>0.05) | No |  |  |
|  |  | 6) Childhood antisocial conduct (10; female)  ->occupational status (30): B=0.007 (p>0.05) | No |  |  |
|  |  | 7) Attention deficit (10; male)  ->employment (30): B=-0.118 (p<0.001) | Yes |  |  |
|  |  | 8) Attention deficit (10; female)  ->employment (30): B=-0.111 (p<0.001) | Yes |  |  |
|  |  | 9) Attention deficit (10; male)  ->lower occupational status (30): B=0.126 (p<0.001) | Yes |  |  |
|  |  | 10) Attention deficit (10; female)  ->lower occupational status (30): B=0.134 (p<0.001) | Yes |  |  |
|  |  | 11) Attention deficit (10; male)  ->lower earnings (30): B=-0.035 (p<0.05) | Yes |  |  |

| Table C. (cont.) | | | | | |
| --- | --- | --- | --- | --- | --- |
| **Reference** | **Study**  **(sample size)** | **Health predictor (age)-> Outcome (age): strength of the association (SE/CI95%)*** | **Associated (Yes/No)** | **Adjusted covariates** | **Quality of evidence (Good/Fair/Poor)** |
| Knapp et al. (2011)  continued | As above | 12) Attention deficit (10; female)->lower earnings (30):  B=-0.087 (p<0.001) | Yes | As above. | As above |
|  |  | 13) Anxiety (10; male)->earnings (30):  B=-0.027 (p>0.05) | No |  |  |
|  |  | 14) Anxiety (10; female)->earnings (30):  B=-0.376 (p>0.05) | No |  |  |
|  |  | 15) Anxiety (10; male)->employment (30):  B=-0.021 (p>0.05) | No |  |  |
|  |  | 16) Anxiety (10; female)->employment (30):  B=-0.039 (p>0.05) | No |  |  |
|  |  | 17) Anxiety (10; male)->lower occupational status (30): B=0.115 (p>0.05) | No |  |  |
|  |  | 18) Anxiety (10; female)  ->lower occupational status (30): B=0.007 (p>0.05) | No |  |  |
|  |  | 19) Physical health problems (10; male)->earnings (30): B=-0.001 (p>0.05) | No |  |  |
|  |  | 20) Physical health problems (10; female)->earnings (30): B=0.029 (p>0.05) | No |  |  |
|  |  | 21) Physical health problems (10; male)->unemployment (30): B=-0.190 (p<0.05) | Yes |  |  |
|  |  | 22) Physical health problems (10; female)->employment (30): B=0.029 (p>0.05) | No |  |  |
|  |  | 23) Physical health problems (10; male)->lower occupational status (30): B=-0.001 (p>0.05) | No |  |  |
|  |  | 24) Physical health problems (10; female)->lower occupational status (30): B=-0.001 (p>0.05) | No |  |  |

| Table D. | | | | | |
| --- | --- | --- | --- | --- | --- |
| **Reference** | **Study**  **(sample size)** | **Health predictor (age)-> Outcome (age): strength of the association (SE/CI95%)*** | **Associated (Yes/No)** | **Adjusted covariates** | **Quality of evidence (Good/Fair/Poor)** |
| Clarke & McKay (2012) | BHPS (913-5082)  LOS (4814-5859) | 1) LOS: disability (30+)->less likely to be married (30+) | Yes | None. | **Poor** |
|  |  | 2) LOS: disability (30+)->more likely to be single (30+) | Yes |  |  |
|  |  | 3) LOS: disability (30+)->more likely to divorce (30+) | Yes |  |  |
|  |  | 4) LOS: disability (30+)->not having children (30+) | Yes |  |  |
|  |  | 5) BHPS: disability (30-69; 1991)->less likely to be married (30-69) | Yes |  |  |
|  |  | 6) BHPS: disability (30-69; 1991)->more likely to be single (30-69) | Yes |  |  |
|  |  | 7) BHPS: disability (30-69; 1991)->not having children (30-69) | Yes |  |  |
|  |  | 8) BHPS: disability (30-69; 1991)->more likely to divorce (30-69) | No |  |  |
| Garcia-Gomez et al. (2010) | BHPS (1991-2002; 2407- 5626) | 1) Health shock as a latent variable (16-64; self-reported general health/health limitations; male)->employment entry less likely: HR=0.406 (0.073) to 1.015 (0.212) | Yes | Employment history; employment of the partner; age; gender; marital status; educational attainment; ethnicity; region of residence; employment skills; reporting bias; mental health; the results are robust to different definitions of employment, and to the exclusion of older workers from the analysis. | **Good** |
|  |  | 2) Health shock as a latent variable (16-64; self-reported general health/health limitations; male)->employment exit more likely: HR=0.681 (0.101) to 0.779 (0.106) | Yes |  |  |
|  |  | 3) Health shock as a latent variable (16-64; self-reported general health/health limitations; female)->employment entry less likely: HR=0.561 (0.068) to 1.059 (0.143) | Yes |  |  |
|  |  | 4) Health shock as a latent variable (16-64; self-reported general health/health limitations; female)->employment exit more likely: HR=0.794 (0.089) to 0.936 (0.105) | Yes |  |  |
| Lindeboom et al. (2016) | NCDS (12375) | 1) Accidents leading to disability (25)->unemployment (40):  B=-0.144 (0.048) | Yes | Birth weight and health; cognitive ability; paternal social class; educational attainment. | **Good** |

| Table D. (cont.) | | | | | |
| --- | --- | --- | --- | --- | --- |
| **Reference** | **Study**  **(sample size)** | **Health predictor (age)-> Outcome (age): strength of the association (SE/CI95%)*** | **Associated (Yes/No)** | **Adjusted covariates** | **Quality of evidence (Good/Fair/Poor)** |
| Lindeboom et al. (2010) | NCDS (7237-8332) | 1) Obesity (33; male)->employment (42):  B=-0.027 (0.015) | No | Demographic variables (e.g. marital status, ethnicity, education); parental background (e.g. permanent income, parental education, financial difficulties); cognitive ability (e.g. test scores on math, reading scores); parental inputs (e.g. going for walks, outings, going to swimming pools, smoking during pregnancy). | **Good** |
|  |  | 2) Obesity (33; female)->unemployment (42):  B=-0.046 (0.022) | Yes |  |  |
|  |  | 3) Parental obesity (acting as an instrument; male) ->employment (42): B=0.149 (0.103) | No |  |  |
|  |  | 4) Parental obesity (acting as an instrument)->  (42; female)->employment (42): B=0.082 (0.176) | No |  |  |
| Manor et al. (2003) | NCDS (8185-8699) | 1) Poor-rated health (23; male)->downward inter-generational mobility (23-33): OR=1.40 (1.03-1.91) | Yes | Prior social class. | **Good** |
|  |  | 2) Poor-rated health (23; female)->downward inter-generational mobility (23-33): OR=1.63 (1.18-2.21) | Yes |  |  |
|  |  | 3) Poor-rated health (23; male)->downward intra-generational mobility (23-33): OR=1.94 (1.43-2.64) | Yes |  |  |
|  |  | 4) Poor-rated health (23; female)->downward intra-generational mobility (23-33): OR=1.65 (1.25-2.16) | Yes |  |  |
| Morris et al. (2006) | HSE (5658) | 1) BMI (18-65; 1997-1998; male)->occupational attainment (earnings) (18-65): B=0.666 (t=2.8) | Yes | Education; health; job characteristics; home and family variables (e.g. marital status and housing tenure); potential work experience; ethnicity rurality; region of residence; HSE year. | **Good** |
|  |  | 2) BMI (18-60; 1997-1998; female)->occupational attainment (earnings) (18-60): B=-0.259 (t=-2.1) | Yes |  |  |
|  |  | 3) Mean BMI in the area (acting as an instrument; male)->lower occupational attainment (earnings) (18-60): B=4.941 (t=0.9) | No |  |  |
|  |  | 4) Mean BMI in the area (acting as an instrument; female)->lower occupational attainment (earnings) (18-60): B=3.080 (t=1.1) | No |  |  |
|  |  | 5) Prevalence of obesity in the area (acting as an instrument; male)->lower occupational attainment (earnings) (18-60): B**=**1.364 (t=0.5) | No |  |  |
|  |  | 6) Prevalence of obesity in the area (acting as an instrument; female)->lower occupational attainment (earnings) (18-60): B=1.245 (t=1.0) | No |  |  |

| Table D. (cont.) | | | | | |
| --- | --- | --- | --- | --- | --- |
| **Reference** | **Study**  **(sample size)** | **Health predictor (age)-> Outcome (age): strength of the association (SE/CI95%)*** | **Associated (Yes/No)** | **Adjusted covariates** | **Quality of evidence (Good/Fair/Poor)** |
| Rahi et al. (2009a/b) | NCDS (9330) | 1) Visual impairment (44/45)->more likely unemployed (44/45): OR=0.87 (0.49-1.54) to 1.74 (0.20-15.4) | No | Gender; social class at 42 years; employment; marital status. | **Fair** |
|  |  | 2) Visual impairment (44/45)->more likely unemployed due to permanent sickness (44/45):  OR=1.53 (1.15-2.03) to 4.63 (2.67-8.03) | Yes |  |  |
|  |  | 3) Visual impairment (44/45)->low socioeconomic status (44/45): OR=1.23 (1.08-1.41) to 2.55 (1.36-4.79) | Yes |  |  |
|  |  | 4) Visual impairment (44/45)->not married (44/45):  OR= 1.10 (0.94-1.28) to 1.67 (1.31-2.12) | Yes |  |  |
|  |  | 5) Visual impairment (44/45)->membership in social and professional organisations (44/45):  OR=0.74 (0.57-0.96) to 1.41 (0.55-3.60) | No |  |  |
|  |  | 6) Visual impairment (44/45)->low quality of life (44/45): OR=2.39 (1.17-4.86) to 10.03 (1.69-59.50) | Yes |  |  |
| Sargent & Blanchflower (1994) | NCDS (12537) | 1) Obesity (23 but not at 16; male)->hourly earnings (23): B=-0.6 (0.2) | No | Social class; IQ; unemployment rate; type of industry; marital status; highest educational qualifications; plant size; part-time dummy; union membership; ethnicity. | **Fair** |
|  |  | 2) Obesity (23 but not at 16; female)->hourly earnings (23): B=-3.0 (0.9) | No |  |  |
| Schick & Steckel (2015) | NCDS (2550) | 1) Height (33; male)->hourly earnings (33):  B=0.005 (0.005) | No | Cognitive math and reading test scores,  reported at age 11; problem-solving skills reported at age 33; emotional stability and extroversion assessment scores reported at ages 11, 16, 23, and 33. | **Fair** |
|  |  | 2) Height (33; female)->hourly earnings (33):  B=0.000 (0.006) | No |  |  |

| Table E. | | | | | |
| --- | --- | --- | --- | --- | --- |
| **Reference** | **Study**  **(sample size)** | **Health predictor (age)-> Outcome (age): strength of the association (SE/CI95%)*** | **Associated (Yes/No)** | **Adjusted covariates** | **Quality of evidence (Good/Fair/Poor)** |
| Chittlebo-rough et al. (2011) | ALSPAC (8159-8265) | 1) Postnatal depression (when child 8 weeks old)-> feelings of poor attachment/hostility (child 47 months old): OR=1.74 (1.38-2.19); 1.63 (1.36-1.94) | Yes | Age; partner status; financial difficulties; smoking in first 3 months of pregnancy; educational attainment. | **Good** |
|  |  | 2) Postnatal depression (when child 8 weeks old)->NEET (child 61 months old): OR=1.10 (0.85-1.42) | No |  |  |
| Johnston et al. (2013) | BCS70 (8194) | 1) Psychological distress (26, 30, 34)->  lower household income (30, 34): B=-0.083 (0.008) | Yes | Maternal mental health; health (age 0/5); family characteristics (age 5); test scores (age 5); mental health (age 5); physical health (age 30); having a degree; maternal education; parental social class; no father in household; vocabulary and ability tests. | **Good** |
| Stansfeld et al. (2007) | NCDS (8243) | 1) Psychological distress (23; 33)->high job demands (45): RR=1.44 (1.13-1.84); 1.58 (1.26-1.99) | Yes | Sex; housing tenure at age 7; marital status at 45; educational attainment at 33; physical ill health at 42; mental health diagnosis at 45. | **Fair** |
|  |  | 2) Psychological distress (23; 33)->low decision latitude (45): RR=1.54 (1.22-1.94); 1.55 (1.20-2.00) | Yes |  |  |
|  |  | 3) Psychological distress (23; 33)->low social support (45): RR=1.39 (1.08-1.78); 1.34 (1.07-1.69) | Yes |  |  |
|  |  | 4) Psychological distress (23; 33)->job insecurity (45): OR=1.44 (1.15-1.80); 1.32 (0.99-1.77) | Yes |  |  |
|  |  | 5) Psychological distress (23; 33)->high job strain (45): OR=1.39 (1.13-1.70); 1.53 (1.24-1.88) | Yes |  |  |
| Woodhead et al. (2015) | Understanding Society (40476) | 1) Psychological distress (16+; 2010-2011)->unmet residential mobility preferences (16+; 2011-2012): RRR=1.22 (0.92-1.64) to 1.42 (1.29-1.56) | Yes | Sex; age; marital status; ethnicity; education; parental status; tenure; employment; benefits receipt; household income; financial situation; overcrowding. | **Good** |

| Table F. | | | | | |
| --- | --- | --- | --- | --- | --- |
| **Reference** | **Study**  **(sample size)** | **Health predictor (age)-> Outcome (age): strength of the association (SE/CI95%)*** | **Associated (Yes/No)** | **Adjusted covariates** | **Quality of evidence (Good/Fair/Poor)** |
| Denny & Doyle (2007) | NCDS (6653-8463) | 1) Psychological distress (23, 33, 42)->turnout in general elections (1979): B=-0.002 (0.002) | No | Region of residence; sex, fathers/mothers social class at birth; overcrowding in household at age 7; three binary variables indicating how long respondent missed school due to illness at age 11, age left full-time education; whether stayed in school beyond 16 and whether the respondent is married; has children; being unemployed or self-employed at the time of each election. | **Fair** |
|  |  | 2) Psychological distress (23, 33, 42)->turnout in general elections (1987): B=-0.005 (0.002) | Yes |  |  |
|  |  | 3) Psychological distress (23, 33, 42)->turnout in general elections (1997): B=-0.002 (0.002) | No |  |  |
|  |  | 4) General health (23, 33, 42)->turnout in general elections (1979): B=-0.043 (0.020) | Yes |  |  |
|  |  | 5) General health (23, 33, 42)->turnout in general elections (1987): B=-0.021 (0.016) | No |  |  |
|  |  | 6) General health (23, 33, 42)->turnout in general elections (1997): B=-0.040 (0.015) | Yes |  |  |
| Henderson et al. (2013) | BCS70 (14105) | 2) Self-rated health (30)->receiving Incapacity Benefit/Severe Disablement Allowance (34):  OR=5.5 (2.3–12.9) | Yes | Adult health variables at age 30, maternal rating of participant’s health at age 16, model, depression/ anxiety/headache/stomach ache all measured at age 16. | **Good** |
| Henderson et al. (2012) | NCDS (11419) | 1) Psychological distress (33)->long term sickness absence (42): OR=2.2 (1.6-3.2) | Yes | Sex, social class at birth; social class aged 33; cognitive ability; highest educational attainment at age 33; decision latitude; musculoskeletal symptoms age 33; "worries" age 11; being "miserable" age 11; headaches age 11; tummy-aches age 11. | **Good** |
|  |  | 2) Musculoskeletal symptoms (33)  ->long-term sickness absence (42): OR=1.3 (1.0-1.8) | Yes |  |  |
| Waylen et al. (2009) | ALSPAC (9687-11314) | 1) Deterioration in general health (8 months; 33 months)->quality of parenting (8 months; 33 months): B=0.03 (-0.06 to 0.12) | No | Changes in financial circumstance, social support, and maternal general health and depression (where appropriate), age, education, and baseline (8 month) depression and parenting score. | **Good** |
|  |  | 2) Increase in depression symptoms (8 months; 33 months)->lower quality of parenting (8 months; 33 months): B=-0.14 (-0.23 to -0.04) | Yes |  |  |

| Table G. | | | | | |
| --- | --- | --- | --- | --- | --- |
| **Reference** | **Study**  **(sample size)** | **Health predictor (age)-> Outcome (age): strength of the association (SE/CI95%)*** | **Associated (Yes/No)** | **Adjusted covariates** | **Quality of evidence (Good/Fair/Poor)** |
| Blane et al. (2008) | ELSA (5383- 5718) | 1) Blood pressure (50+; 2002-2003; male)  ->quality of life (52+; 2004-2005):  B=-0.02 (-0.06 to 0.02); -0.02 (-0.04 to 0.00) | No | Baseline measurements and age, age2, the differences in number of years since Wave 0; tested for mediators (functional limitations and depression) showing their effect. | **Good** |
|  |  | 2) Lung problem (50+; 2002-2003; male)  -> lower quality of life (52+; 2004-2005):  B=0.84 (0.04 to 1.65); 1.48 (0.35 to 2.60) | Yes |  |  |
|  |  | 3) BMI (50+; 2002-2003; male)  ->quality of life (52+; 2004-2005):  B=-0.11 (-0.33 to 0.12) | No |  |  |
|  |  | 4) Blood pressure (50+; 2002-2003; female)  ->quality of life (52+; 2004-2005):  B=-0.02 (-0.05 to 0.02); -0.01 (-0.03 to 0.01) | No |  |  |
|  |  | 5) Lung problem (50+; 2002-2003; female)  ->lower quality of life (52+; 2004-2005):  B=1.51 (0.50 to 2.52); 1.67 (0.16 to 3.19) | Yes |  |  |
|  |  | 6) Higher BMI (50+; 2002-2003; female)  ->lower quality of life (52+; 2004-2005):  B=-0.28 (-0.43 to -0.12) | Yes |  |  |
| Candon (2015) | ELSA (11496) | 1) Any type of cancer (50-67)-> unemployment (6,12,18, 24 months after diagnosis): B=-0.119 (0.037) | Yes | Age, sex, education, marital status, family status, general health, smoking, alcohol consumption, earnings, being self-employed, having physical job. | **Fair** |
|  |  | 2) Any type of cancer (50-67)->lower number of hours worked per week (6,12,18, 24 months after diagnosis): B=-4.692 (1.236) | Yes |  |  |

| Table G. (cont.) | | | | | |
| --- | --- | --- | --- | --- | --- |
| **Reference** | **Study**  **(sample size)** | **Health predictor (age)-> Outcome (age): strength of the association (SE/CI95%)*** | **Associated (Yes/No)** | **Adjusted covariates** | **Quality of evidence (Good/Fair/Poor)** |
| Disney et al. (2006) | BHPS (1991-1998; 962) | 1) Health shock as a latent variable (50-64; 1991)  ->early retirement (58-64; 1998):  B=-0.583 (0.128) to -0.577 (0.106) | Yes | Educational achievement; family composition; region of residence; housing wealth. | **Good** |
| Frijters et al. (2014) | NCDS (4400) | 1) Poor health (50)->lower life satisfaction (50):  B=-0.661 (-11.89) | Yes | Age 7: test scores for arithmetic, copying design, draw-a-man and reading, chronic health conditions, an index of behavioural problems and the BSMG score; updated measures of father’s occupational class and dummy indicators for health; Age 11/16: health, cognitive and non-cognitive attributes, number of siblings, whether or not the biological father is in the household and father’s occupational status. | **Fair** |
| Jivraj et al. (2016) | ELSA (5315) | 1) Poor self-reported general health (52+; 2002-2010; male)->subsequent social detachment (52+; 2002-2010): RRR=1.48 (1.19-1.83) | Yes | Age; education; household wealth; marital status; employment status; place of residence; access to a car. | **Good** |
|  |  | 1) Poor self-reported general health (52+; 2002-2010; female)->subsequent social detachment (52+; 2002-2010): RRR=1.37 (1.10-1.69) | Yes |  |  |
| Jivraj & Nazroo (2014) | ELSA (6733) | 1) Having chronic conditions (55+)->lower life satisfaction (55+; 2004): B=-2.043 (0.257) | Yes | Age; marital status; wealth; education. | **Poor** |
|  |  | 2) Having disability (55+)->lower quality of life (55+; 2004): B=-7.488 (0.421) | Yes |  |  |
|  |  | 3) Having chronic conditions (55+)->lower quality of life (55+; 2004): B=-2.825 (0.297) | Yes |  |  |

| Table G. (cont.) | | | | | |
| --- | --- | --- | --- | --- | --- |
| **Reference** | **Study**  **(sample size)** | **Health predictor (age)-> Outcome (age): strength of the association (SE/CI95%)*** | **Associated (Yes/No)** | **Adjusted covariates** | **Quality of evidence (Good/Fair/Poor)** |
| Jones et al. (2010) | BHPS (1991-2002; 1135) | 1) Latent health shocks (50-64; 1991-2002; male)  ->earlier retirement (50-64; 1991-2002):  B=4.895 (2.064) | Yes | Employment history; employment of the partner; age; gender; marital status; educational attainment; ethnicity; region of residence; employment skills; reporting bias; private pension entitlement; sector of employment. | **Good** |
|  |  | 2) Poor self-reported health (50-64; 1991-2002; male)->earlier retirement (50-64; 1991-2002): B=3.908 (0.939) | Yes |  |  |
|  |  | 3) Latent health shocks (50-64; 1991-2002; female)  ->earlier retirement (50-64; 1991-2002):  B=7.223 (4.288) | Yes |  |  |
|  |  | 4) Poor self-reported health (50-64; 1991-2002; female)->earlier retirement (50-64; 1991-2002): B=5.780 (2.108) | Yes |  |  |
| Matthews et al. (2017) | ELSA (28086) | 1) Deterioration in self-reported vision (50+; between 2002-2003 and 2012-2013)->lower life satisfaction (following change in vision): B=-0.38 (p>0.05) | Yes | Gender; age group; baseline outcome and baseline vision; ethnicity; wealth; perceived social status; employment status; self-reported health. | **Fair** |
|  |  | 2) Deterioration in self-reported vision (50+; between 2002-2003 and 2012-2013)->lower quality of life (following change in vision): B=-1.04 (p<0.001) | Yes |  |  |
|  |  | 3) Deterioration in self-reported vision (50+; between 2002-2003 and 2012-2013)->social disengagement (following change in vision): B=0.03 (p<0.05) | Yes |  |  |
|  |  | 4) Deterioration in self-reported vision (50+; between 2002-2003 and 2012-2013)->equivalised income (following change in vision): B=-9.27 (p>0.05) | No |  |  |
| Rouxel et al. (2015) | ELSA (11391) | 1) Poor self-rated oral health (50+; 2006-07)->  lower functional social capital (54+; 2010-11):  RRR=1.02 (0.80-1.30) to 1.39 (1.14-1.70) | Yes | Demographic, socio-economic, health-related factors, smoking status, and baseline outcome variable. | **Fair** |
|  |  | 2) Edentate status (50+; 2006-07)->  lower functional social capital (54+; 2010-11):  RRR=0.88 (0.65-1.19) to 1.34 (0.99-1.81) | No |  |  |
| Rumball-Smith et al. (2014) | ELSA (11096) | 1) Diabetes (50+; 2002, 2004, and 2006)->labour force exit (50+; 2002, 2004, and 2006):  HR=1.14 (0.94-1.39) | No | Year of birth; sex; education. | **Poor** |

| Table G. (cont.) | | | | | |
| --- | --- | --- | --- | --- | --- |
| **Reference** | **Study**  **(sample size)** | **Health predictor (age)-> Outcome (age): strength of the association (SE/CI95%)*** | **Associated (Yes/No)** | **Adjusted covariates** | **Quality of evidence (Good/Fair/Poor)** |
| Stafford et al. (2017) | NSHD (2452-2661) | 1) Number of reported physical limitations (53)  ->more likely to retire before state pension age for negative reasons (60-64; 68):  RRR (3-4 limitations vs none)=3.15 (1.07-9.26) | Yes | Occupational class; education; partner’s employment; work disability at age 53; gender. | **Good** |
|  |  | 2) Number of reported physical limitations (53)  ->less likely to participate in bridge employment  (60-64; 68):  RRR (3-4 limitations vs none)=0.39 (0.14-1.06) | Yes |  |  |
|  |  | 3) Performance-based physical capability (53)  ->likely to retire before state pension age for negative reasons (60-64; 68):  RRR (bottom 10%)=1.01 (0.63-1.62) | No |  |  |
|  |  | 4) Performance-based physical capability (53)->less likely to participate in bridge employment (60-64; 68): RRR (bottom 10%)=0.65 (0.40-1.04) | Yes |  |  |
| Williams et al. (2016) | ELSA (444-4564) | 1) Any type of cancer (50+; 2002-2012)->lower quality of life (0-2; 2-4 years post-diagnosis): p<0.001; estimates not available | Yes | Age; gender; wealth. | **Poor** |
|  |  | 2) Any type of cancer (50+; 2002-2012)->lower life satisfaction (0-2; 2-4 years post-diagnosis): p<0.01; estimates not available | Yes |  |  |
| Wikman et al. (2011) | ELSA (11523) | 1) Chronic condition (cancer, osteoarthritis, asthma, diabetes, coronary heart disease, rheumatoid arthritis, chronic lung disease, stroke; 50+)->lower quality of life (50+):  B=37.56 (36.73 to 38.39) to 41.68 (41.03 to 42.33) | Yes | Age; gender; wealth. | **Poor** |

| Table G. (cont.) | | | | | |
| --- | --- | --- | --- | --- | --- |
| **Reference** | **Study**  **(sample size)** | **Health predictor (age)-> Outcome (age): strength of the association (SE/CI95%)*** | **Associated (Yes/No)** | **Adjusted covariates** | **Quality of evidence (Good/Fair/Poor)** |
| Trevisan & Zantomio (2016) | ELSA (11375) | 1) Acute health shock (the first onset of myocardial infarction, stroke or cancer; 50+; 2002/3-2004/5; male)->lower labour market participation (50+; 2002/3-2004/5): Average Treatment effect on the Treated (ATT)=-0.101 (0.026) | Yes | Gender; age; time; country; education; household size; number of children; income; home owner; health related limitations; self-assessed health; death of parents; high blood pressure; smoking; disability; earnings; expects early retirement due to health. | **Fair** |
|  |  | 2) Acute health shock (the first onset of myocardial infarction, stroke or cancer; 50+; 2002/3-2004/5; male)->higher number of hours worked per week (50+; 2002/3-2004/5): ATT=4.075 (1.272) | Yes |  |  |
|  |  | 3) Acute health shock (the first onset of myocardial infarction, stroke or cancer; 50+; 2002/3-2004/5; female)->lower labour market participation (50+; 2002/3-2004/5): ATT=-0.130 (0.031) | Yes |  |  |
|  |  | 4) Acute health shock (the first onset of myocardial infarction, stroke or cancer; 50+; 2002/3-2004/5; female)->number of hours worked per week (50+; 2002/3-2004/5): ATT=0.974 (1.732) | No |  |  |
| Zaninotto et al. (2016) | ELSA (4996) | 1) Coronary heart disease (50+; 2002-03 to 2006-07; male)->lower quality of life (50+; 2002-03 to 2006-07): B~=1.4 | Yes | Gender; age; cohabiting status; employment status; educational attainment; non-pension wealth; smoking status; alcohol consumption; physical activity; whether or not often troubled with pain, one or more ADLs; social support; number of close friends; baseline quality of life. | **Good** |
|  |  | 2) Coronary heart disease (50+; 2002-03 to 2006-07; female)->lower quality of life (50+; 2002-03 to 2006-07): B~=1.1 | Yes |  |  |

| Table G. (cont.) | | | | | |
| --- | --- | --- | --- | --- | --- |
| **Reference** | **Study**  **(sample size)** | **Health predictor (age)-> Outcome (age): strength of the association (SE/CI95%)*** | **Associated (Yes/No)** | **Adjusted covariates** | **Quality of evidence (Good/Fair/Poor)** |
| Zaninotto et al. (2010) | ELSA (8688) | 1) Waist circumference (52+; 2004-2005; male)->quality of life (54+; 2006-2007): B=0.19 (-0.07 to 0.45) | No | Age; cohabiting; wealth; limiting long-standing illness; ADLs; alcohol consumption; smoking; quality of life at baseline; BMI and waist circumference where appropriate. | **Good** |
|  |  | 2) Waist circumference (52+; 2004–2005; female)-> quality of life (54+; 2006-2007):  B=-0.12 (-0.32 to 0.08) | No |  |  |
|  |  | 3) BMI (52+; 2004-2005; male)->quality of life (54+; 2006–2007): B=-0.17 (-0.48 to 0.13) | No |  |  |
|  |  | 4) BMI (52+; 2004-2005; female)->quality of life (54+; 2006–2007): B=0.08 (-0.13 to 0.28) | No |  |  |
|  |  | 5) Having a limited long-standing illness (52+; 2004-2005; male)->worse quality of life (54+; 2006-2007):  B=-1.39 (-2.01 to -0.78) | Yes |  |  |
|  |  | 6) Having a limited long-standing illness (52+; 2004-2005; female)->worse quality of life (54+; 2006-2007): B=-0.58 (-1.12 to -0.03) | Yes |  |  |
|  |  | 7) Having ADL (52+; 2004-2005; male)->quality of life (54+; 2006-2007): B=-0.71 (-1.50 to 0.09) | No |  |  |
|  |  | 8) Having ADL (52+; 2004–2005; female)->worse quality of life (54+; 2006-2007): B=-1.09 (-1.72 to -0.45) | Yes |  |  |

| Table H. | | | | | |
| --- | --- | --- | --- | --- | --- |
| **Reference** | **Study**  **(sample size)** | **Health predictor (age)-> Outcome (age): strength of the association (SE/CI95%)*** | **Associated (Yes/No)** | **Adjusted covariates** | **Quality of evidence (Good/Fair/Poor)** |
| Nishida et al. (2016) | NSHD (1561) | 1) Psychological distress (60-64)->lower life satisfaction (60-64): B=-0.493 (p<0.001) | Yes | Emotional problems/ conduct problems/ self-regulation (where appropriate); gender; childhood cognitive ability at age 8; father’s occupational social class; educational attainment by age 26; GHQ-28 total score at age 60–64. | **Fair** |

| Table I. | | | | | |
| --- | --- | --- | --- | --- | --- |
| **Reference** | **Study**  **(sample size)** | **Health predictor (age)-> Outcome (age): strength of the association (SE/CI95%)*** | **Associated (Yes/No)** | **Adjusted covariates** | **Quality of evidence (Good/Fair/Poor)** |
| Rice et al. (2011) | ELSA (308- 1693) | 1) Depression (50-56/61; 2002)  ->early retirement (under 60 or 65): OR=1.50 (1.06-2.15) | Yes | Wealth; partner retirement (whether respondents had a partner who retired during the follow-up period); education; activity level of job; smoking status; alcohol consumption frequency; body mass index; self-rated health. | **Good** |
|  |  | 2) Mobility limitations, mainly lower limb pain and shortness of breath (50-56/61; 2002)->early retirement (under 60 or 65):  OR=1.04 (0.76-1.42) to 19.8 (5.67-68.9) | Yes |  |  |
|  |  | 3) Multiple poor health symptoms (50-56/61; 2002)->early retirement (under 60 or 65): OR=1.71 (1.20-2.44) | Yes |  |  |
|  |  | 4) Poor self-rated health (50-56/61; 2002)->early retirement (under 60 or 65): OR=2.14 (p<0.001) | Yes |  |  |
|  |  | 5) Self-reported longstanding limiting illness (50-56/61; 2002)  ->early retirement (under 60 or 65): OR=1.73 (p<0.001) | Yes |  |  |
|  |  | 6) Having IADLs (50-56/61; 2002)  ->early retirement (under 60 or 65): OR=1.96 (p=0.011) | Yes |  |  |
|  |  | 7) Having ADLs (50-56/61; 2002)  ->early retirement (under 60 or 65): OR=1.45 (p=0.138) | No |  |  |
|  |  | 8) Obesity (50-56/61; 2002)  ->early retirement (under 60 or 65): OR=1.21 (p=0.303) | No |  |  |
|  |  | 9) High blood pressure (50-56/61; 2002)  ->early retirement (under 60 or 65): OR=0.93 (p=0.633) | No |  |  |
|  |  | 10) Having a heart problem (50-56/61; 2002)  ->early retirement (under 60 or 65): OR=1.32 (p=0.167) | No |  |  |
| Webb et al. (2011) | ELSA (4848) | 1) Worsening in depression (50+; 2002)  ->deterioration in quality of life (between 2002 and 2006):  B=-3.46 (-4.02 to -2.90) | Yes | Demographics, baseline quality of life, economic circumstances, social contact and relationships, marital status at baseline, neighbourhood. | **Fair** |
|  |  | 2) Worsening in limiting long-standing illness (50+; 2002)  ->deterioration in quality of life (between 2002 and 2006):  B=-1.59 (-2.16 to -1.01) | Yes |  |  |
|  |  | 3) Worsening in long-standing illness (50+; 2002)  ->deterioration in quality of life (between 2002 and 2006):  B=-1.00 (-1.54 to -0.45) | Yes |  |  |
|  |  | 4) Worsening in ADLs (50+; 2002)->deterioration in quality of life (between 2002 and 2006): B=-0.54 (-0.69 to -0.40) | Yes |  |  |

| Table I. (cont.) | | | | | |
| --- | --- | --- | --- | --- | --- |
| **Reference** | **Study**  **(sample size)** | **Health predictor (age)-> Outcome (age): strength of the association (SE/CI95%)*** | **Associated (Yes/No)** | **Adjusted covariates** | **Quality of evidence (Good/Fair/Poor)** |
| Zaninotto et al. (2009) | ELSA (7114-11392) | 1) Depression (50+; between 2002-2003 and 2006-2007)->lower quality of life (50+; between 2002-2003 and 2006–2007):  B=-1.37 (-1.46 to -1.29) to -1.30 (-1.39 to -1.20) | Yes | Symptoms of depression; wealth; employment status; cohabiting status; number of close friends/family; positive social support. | **Good** |
|  |  | 2) Limiting long-standing illness (50+; between 2002-2003 and 2006-2007)->lower quality of life (50+; between 2002-2003 and 2006-2007):  B=-2.93 (-3.23 to -2.63) to -2.26 (-2.62 to -1.89) | Yes |  |  |
|  |  | 3) Difficulty with ADLs (50+; between 2002-2003 and 2006-2007)->lower quality of life (50+; between 2002-2003 and 2006-2007):  B=-1.25 (-1.45 to -1.06) to -0.99 (-1.18 to -0.79) | Yes |  |  |
| ***Note.*** ALSPAC = Avon Longitudinal Study of Parents and Children; MVPA = moderate-to-vigorous physical activity; NCDS = National Child Development Study; SES = socioeconomic status; BCS70 = British Cohort Study; MCS = Millennium Cohort Study; ICD10 = International Statistical Classification of Diseases and Related Health Problems; BMI = body mass index; NSHD = National Survey of Health and Development; LSYPE = Longitudinal Studies of Young People; NEET = Not in Education, Employment, or Training; MID = Mild Intellectual Disability; OLS = ordinary least squares; BSMG = the British Social Maladjustment Guide; GHQ-28 = General Health Questionnaire; HSE = Health Survey for England; GCSE = General Certificate of Secondary Education; ADHD = attention-deficit hyperactivity disorder; DBD = disruptive behaviour disorder; NVQ = National Vocational Qualifications; BHPS = British Household Panel Survey; LOS = Life Opportunities Survey; ELSA = English Longitudinal Study of Ageing; ADL = activities of daily living; IADL = instrumental activities of daily living.  Measures of association: B = beta coefficient; OR = odds ratio; RRR = relative risk ratio; HR = hazard ratio.  *SE/CI95% = standard error/95% confidence interval for association measures provided, unless unavailable.  Associations (between a predictor and outcome) were extracted from the most adjusted models (if more than one model reported). We included a range of values if there was more than one measure of a predictor and/or outcome provided in the study. In cases where results based on different measures were inconsistent (e.g. some measures showed an association and others did not), the overall conclusion about the association between predictor and outcome was based on the conclusion of the author(s). The direction of the association is provided qualitatively (e.g. ‘poor mental health -> not married’ rather than ‘mental health state -> marriage’) and the corresponding association measure should be interpreted accordingly. | | | | | |
