# Supplementary material for: The impact of health on economic and social outcomes in the United Kingdom: A scoping literature review
Source: PLoS One. 2018 Dec 31;13(12):e0209659. doi: 10.1371/journal.pone.0209659 (PMC6312330; doi:10.1371/journal.pone.0209659)
Supplement: S1 Text — (DOCX) [file pone.0209659.s005.docx]

S1 Text.

**Using longitudinal data to explore the impact of health on economic and societal outcomes: a literature review.**

This proposal from the Centre for Longitudinal Studies sets out the aims and methods for this review.

Aims:

This work has two key aims:

1. To explore how British birth cohort and other longitudinal data have been used to date in exploring relationships between the health of individuals, and their economic and social outcomes, specifically focussing on three key areas, namely:
2. the impact of ill health during childhood or teenage years on outcomes across the life course;
3. the impact of ill health in middle age;
4. the impact of ill health on detachment from the labour market at older ages.
5. To understand the further potential for the British birth cohorts, other cohort studies and key longitudinal datasets to be used to explore the impact of the health of individuals on economic and societal outcomes.

Methods & data sources

The work proposed consists of a set of desk research as follows:

- A rapid review of the literature to understand how British birth cohorts and other longitudinal data have been used to date in exploring the impact of the health of individuals on economic and societal outcomes, focusing the size, consistency and quality of the evidence. This rapid review will consist of

- an assessment of clear gaps in the literature where further research exploring the impact of the health of individuals on economic and societal outcomes would be valuable;

- an assessment of the key methodological issues or challenges (for example related to causal inference, missing data, or measurement error) that researchers would need to address order to undertake these analyses.

Rapid review methods:

The first stage of the rapid review is to clearly define the research questions for review.

In terms of the over-arching research question of interest, we propose that the review will be limited to literature addressing the impact of individual health on a number of clearly defined social and economic outcomes. The focus will be on health at three different life stages, namely (i) the impact of ill health during childhood or teenage years on outcomes across the life course; (ii) the impact of ill health in middle age; (iii) the impact of ill health on detachment from the labour market at older ages.

The exposure - ‘health’ - is broadly defined, based on measures of health that have been typically used in the UK-based longitudinal studies. Both physical and mental health outcomes are considered. Physical health exposures include measures of general health, disability and functional limitations, limitations of body systems, infectious disease, chronic health. Mental heal measures include psychological distress, internalising and externalising symptoms and a range of specific mental disorders, such as depression, anxiety or psychosis.

The review will consider the following economic and societal outcomes:

- Social factors:

− Partnership status

− Family

− Social network

− Social capital

− Life satisfaction

− Educational attainment

- Economic factors:

- Employment

- Income

− Wealth

Data sources: The rapid review will not comprehensively cover all literature addressing these questions based on all data sources, but instead will mainly consider literature based on evidence from the UK which uses a number of key data sources to address these questions.

This will consist of the cohort studies managed within the Centre for Longitudinal studies, namely the Millennium Cohort Study (born 2000/1), Next Steps (born 1989/90), the 1970 British Cohort Study (born 1970) and the National Child Development Study (born 1958) in conjunction with a limited number of other UK cohorts used in this field, namely MRC’s National Survey of Health and Development (born in 1946), the English Longitudinal Study of Ageing (ELSA – a representative sample of the 50+ population in England), and Understanding Society (a population representative national panel, and its predecessor the British Household Panel Study).

The work will also consider major substantive outputs on this topic using international data, such as the US Panel Study of Income Dynamics, and the Health and Retirement Study, and the New Zealand Dunedin Study, however the international element will be less comprehensive than the UK aspect.

Evidence consulted: In general rapid reviews take as their first source of evidence relevant existing systematic reviews, or other high quality reviews of either experimental studies or rigorous quantitative studies using observational data. Given the likely paucity of existing reviews covering these research questions (which can rarely be addressed using experimental evidence and thus are not typically covered by systematic reviews) we will conduct our own literature search to individually review high quality studies that are not covered in other reviews. We will mainly focus on high impact factor journal publications and highly cited studies. However all available literature will be considered regardless of publication status, and including grey literature.

Citations will be identified, screened, and synthesised based on the research questions above. In large part the synthesis will be based on abstracts only, though for a subset the retrieval and synthesis of full-text documents will be undertaken. No formal statistical meta-analysis will be conducted. Instead our review will provide an overview of the evidence identified with the goal of providing a sense of the volume, consistency and quality of available evidence addressing the topic of interest.

We will produce a set of evidence summaries, which will extract the primary objective, methods, results and relevant limitations from each included review or primary study. Most studies included in our review are likely to derive from observational data, and we will only select studies on which a rigorous quantitative analysis has been performed, for example using multivariate regressions or quasi-experimental methods, and we will specifically comment on the rigour of the methods used.
